# Supplementary material for: Modelling POLG mutations in mice unravels a critical role of POLγΒ in regulating phenotypic severity
Source: Nat Commun. 2025 May 23;16:4782. doi: 10.1038/s41467-025-60059-y (PMC12098916; doi:10.1038/s41467-025-60059-y)
Supplement: Supplementary file 1 — Supplementary Information [file 41467_2025_60059_MOESM1_ESM.pdf]

Supplementary Information

Modelling POLG mutations in mice unravels a critical role of POLγB in regulating phenotypic severity

Samantha Corrà[1*], Alessandro Zuppardo[1,3*], Sebastian Valenzuela[2*,] Louise Jenninger[2*], Raffaele Cerutti[1,3], Sirelin Sillamaa[2], Emily Hoberg[2], Katarina A.S. Johansson[2], Urska Rovsnik[2], Sara Volta[1,3], Pedro Silva-Pinheiro[4], Hannah Davis[5], Aleksandra Trifunovic[6], Michal Minczuk[5,7], Claes M Gustafsson[2], Anu Suomalainen[8,9], Massimo Zeviani[10, 11], Bertil Macao[2], Xuefeng Zhu [2,12,13#], Maria Falkenberg[2#], Carlo Viscomi[1,3#],

[1]Veneto Institute of Molecular Medicine (VIMM), via Orus 2-35129 Padova, Italy

[2]Department of Medical Biochemistry and Cell Biology, University of Gothenburg, Medicinaregatan 9A P.O. Box 440, 41390 Gothenburg, Sweden

[3]Department of Biomedical Sciences, University of Padova, via Ugo Bassi 58/B-35131 Padova, Italy

[4]MRC Mitochondrial Biology Unit, University of Cambridge, Hills Road, CB2 0XY Cambridge, UK

[5]The Mary Lyon Centre, MRC Harwell, Becquerel Ave, Didcot OX11 0RD, Oxfordshire, UK

[6]Institute for Mitochondrial Diseases and Aging, Faculty of Medicine, CECAD Research Center, 50931, Cologne, Germany.

[7]Department of Clinical Neurosciences, University of Cambridge, CB2 2PY Cambridge, UK

[8]Research Programs Unit, Stem Cells and Metabolism, Biomedicum-Helsinki, Haartmaninkatu 8, University of Helsinki, 00290, Helsinki, Finland.

[9] HUSlab, Helsinki University Hospital, University of Helsinki, 00290, Helsinki, Finland.

[10] Department of Neurosciences, University of Padova, via Belzoni 160, 35121 Padova, Italy

[11]Institute for Maternal and Child Health, IRCCS "Burlo Garofolo", via Istria 61, 34137 Trieste, Italy

[12]School of Basic Medical Sciences, North China University of Science and Technology, Tangshan 063210, China

[13]National Key Laboratory for Development and Utilization of Forest Food Resources, Zhejiang A&F University, Hangzhou 311300, China

*These authors equally contributed to this work

[#]Correspondence to:

Carlo Viscomi

Veneto Institute of Molecular Institute (VIMM)

via Orus 2-35129 Padova, Italy

email: carlo.viscomi@unipd.it

OR

Maria Falkenberg

Department of Medical Biochemistry and Cell Biology,

University of Gothenburg,

Medicinaregatan 9A P.O. Box 440,

SE405 30 Gothenburg, Sweden

email: maria.falkenberg@medkem.gu.se

OR

Xuefeng Zhu

Department of Medical Biochemistry and Cell Biology,

University of Gothenburg,

Medicinaregatan 9A P.O. Box 440,

SE405 30 Gothenburg, Sweden

email: xuefeng.zhu@gu.se

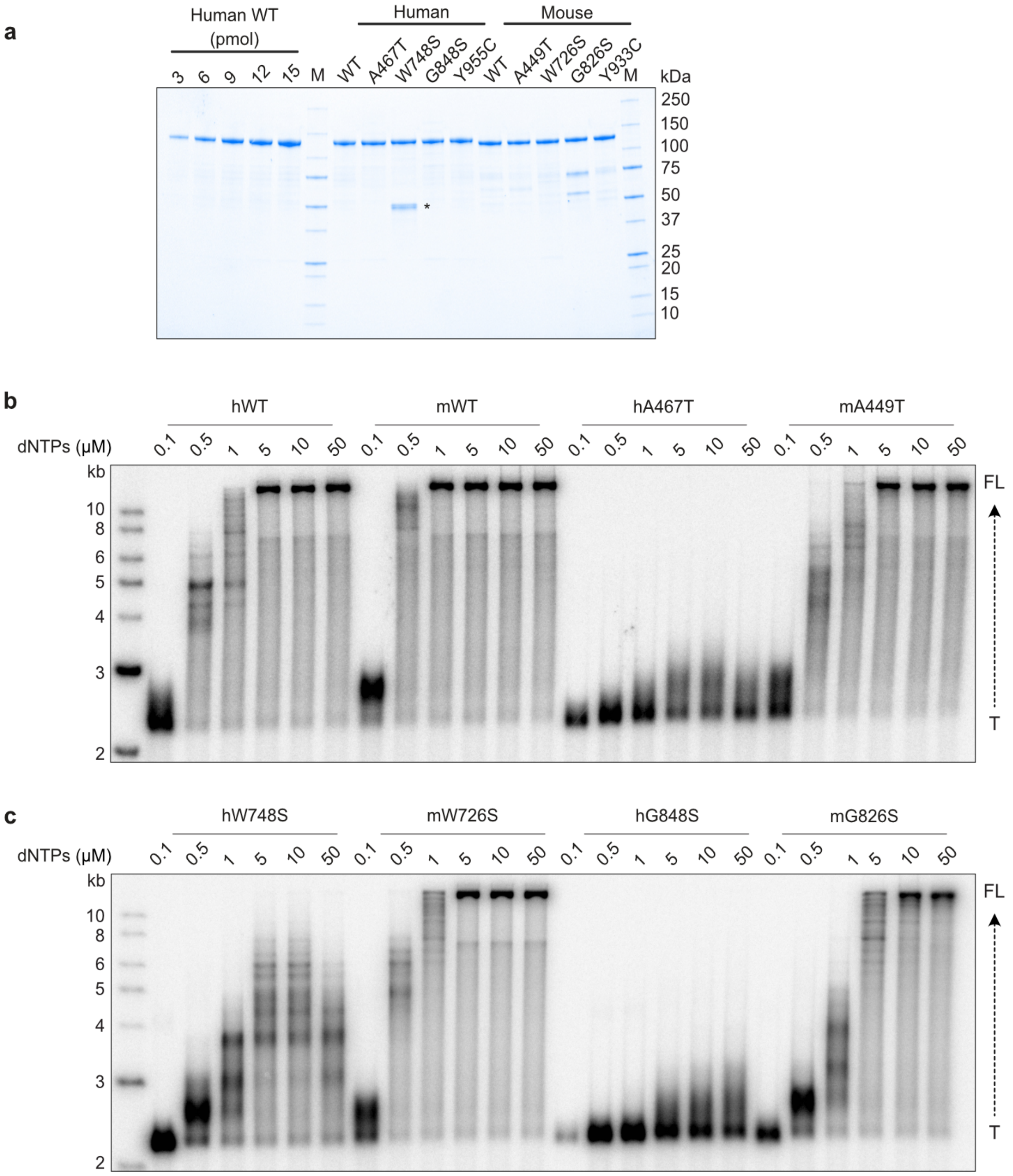

Supplementary Fig. 1. Mutations impair polymerase activity across all tested dNTP concentrations, demonstrating mtSSB interchangeability.

a. Protein gel (SDS-PAGE) assessing the accuracy of POLγ variant quantifications used in enzymatic assays. hPOLγ^{WT} (3–15 pmol) was included as a standard, with 9 pmol serving as a reference. Equal amounts (9 pmol) of human and mouse POLγ variants, based on prior quantification, were loaded for comparison. The asterisk (*) indicates hPOLγB, which was co-expressed with the human hPOLγ^{W748S}. *n* = 2 independent experiments.

b-c. Polymerase activity assays were performed as described in Figure 1**b-c**, but using mouse mtSSB instead of human mtSSB. The results showed a similar pattern, confirming that the choice of mtSSB did not significantly affect polymerase activity. $n = 2$ independent experiments.

Source data are provided as a Source Data file.

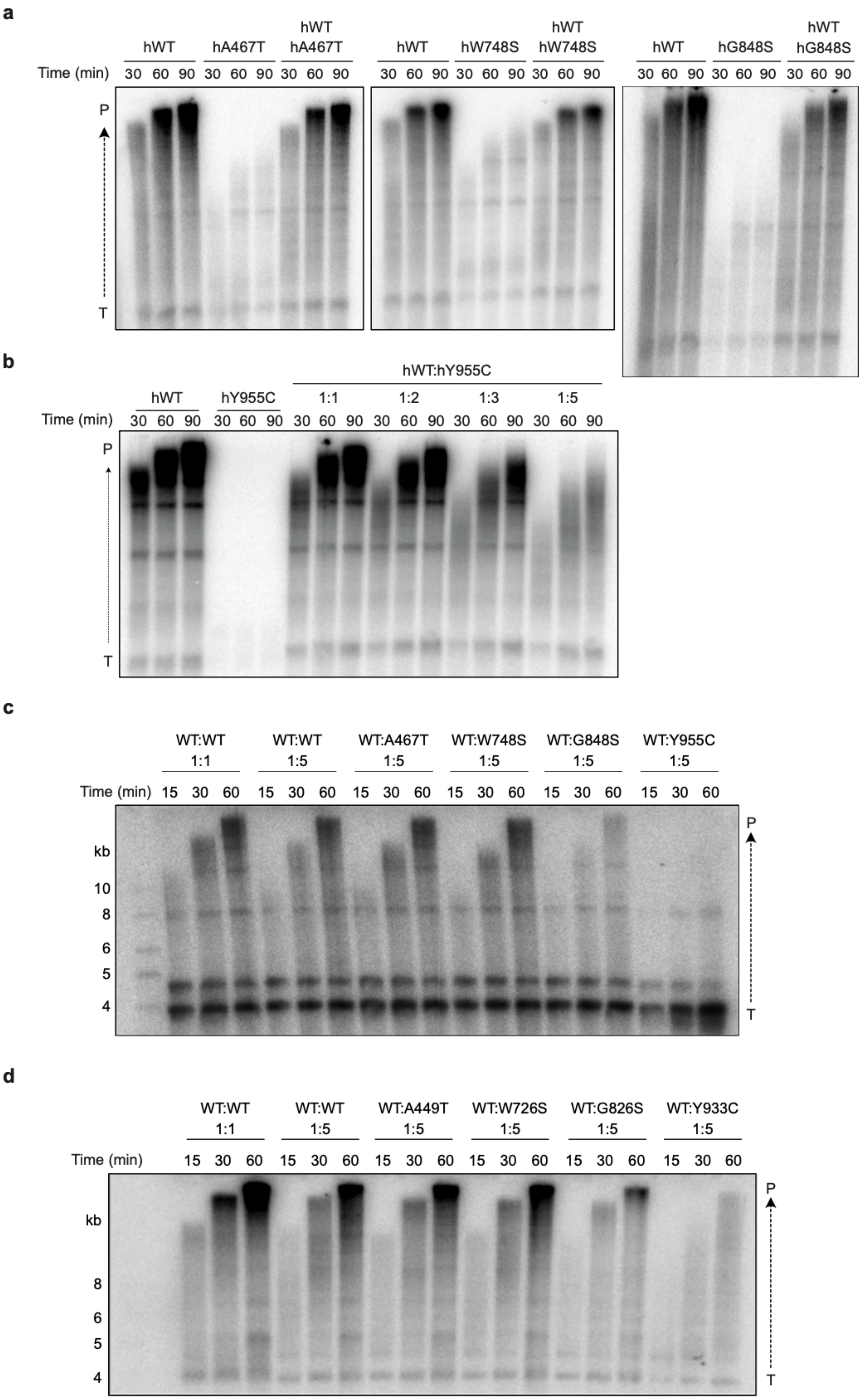
a
hWT
hA467T
hWT hA467T
hWT
hW748S
hWT hW748S
hWT
hG848S
hWT hG848S
Time (min)
30 60 90
P
T
b
hWT:hY955C
hWT
hY955C
1:1
1:2
1:3
1:5
Time (min)
30 60 90
P
T
c
WT:WT 1:1
WT:WT 1:5
WT:A467T 1:5
WT:W748S 1:5
WT:G848S 1:5
WT:Y955C 1:5
Time (min)
15 30 60
kb
10
8
6
5
4
P
T
d
WT:WT 1:1
WT:WT 1:5
WT:A449T 1:5
WT:W726S 1:5
WT:G826S 1:5
WT:Y933C 1:5
Time (min)
15 30 60
kb
8
6
5
4
P
T

Supplementary Fig. 2. The recessive mutations affect the mitochondrial human replisome

a. Time-course experiments performed as outlined in Figure 2a show that replication is compromised with all mutant forms of hPOLγ variants when run in homozygous state during TWINKLE helicase-dependent rolling-circle DNA replication. hPOLγ^{WT} or mutant hPOLγ variants, as indicated, were incubated with TWINKLE, mtSSB, and the rolling-circle template at 37°C for the indicated time points. The polymerase concentration was kept constant; mixed variants were each used at half the concentration to maintain the total enzyme level. DNA synthesis was monitored via radioactive nucleotides incorporation, and the products of increasing length were separated on a 0.8% alkaline agarose gel. Template (T); Products (P). *n* = 2 independent experiments.

b. Time-course experiments, performed as outlined in Figure 2d but with hPOLγ, show that the hPOLγ^{Y955C} variant is unable to synthesis DNA efficiently. When combined with hPOLγ^{WT} at a 1:1 ration, its dominant-negative effect becomes evident and intensifies as the proportion of hPOLγ^{Y955C} increases. *n* = 2 independent experiments.

c. To assess whether recessive mutations exhibit a dominant-negative effect, we performed an additional experiment following the same setup as in **b** but only at the highest ratio (1:5). hPOLγ^{WT} was kept constant, and an excess of either a recessive variant, hPOLγ^{WT}, or the dominant mutant hPOLγ^{Y955C} was added. Adding hPOLγ^{WT}, hPOLγ^{A467T}, or hPOLγ^{W748S} did not reveal any negative effect. However, a weak dominant-negative effect was observed with hPOLγ^{G848S}, although not as severe as that of the dominant hPOLγ^{Y955C} mutant. *n* = 2 independent experiments.

d. The same experiment as in c, showing similar results with mPolγ variants. *n* = 2 independent experiments.

Source data are provided as a Source Data file.

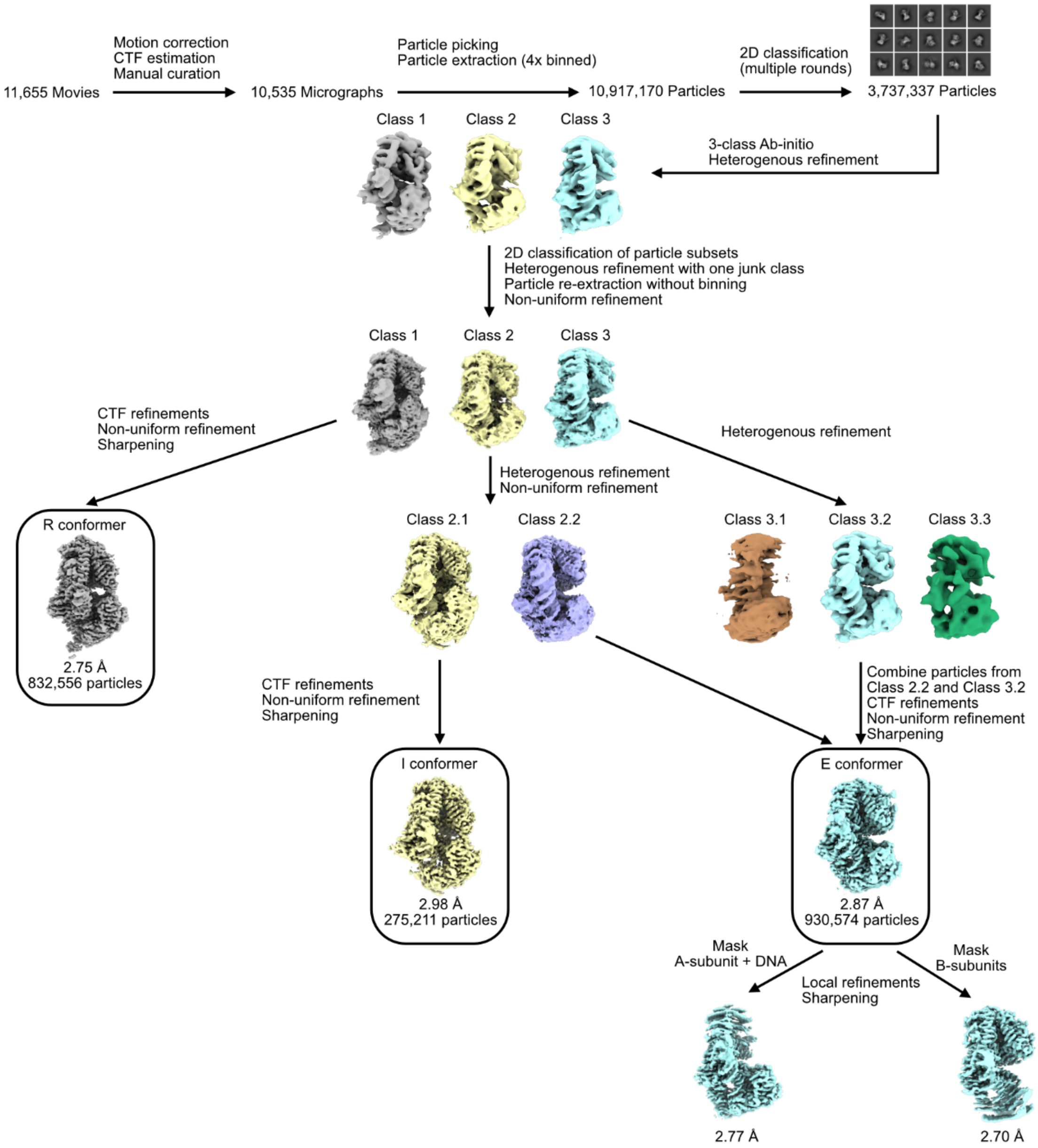

Supplementary Fig. 3. Cryo-EM processing workflow for mPolγ.

Cryo-EM processing workflow for mPolγ. The data were imported into cryoSPARC, which was used for processing and generating the 3D volumes of the three conformers (R, I and E conformers).

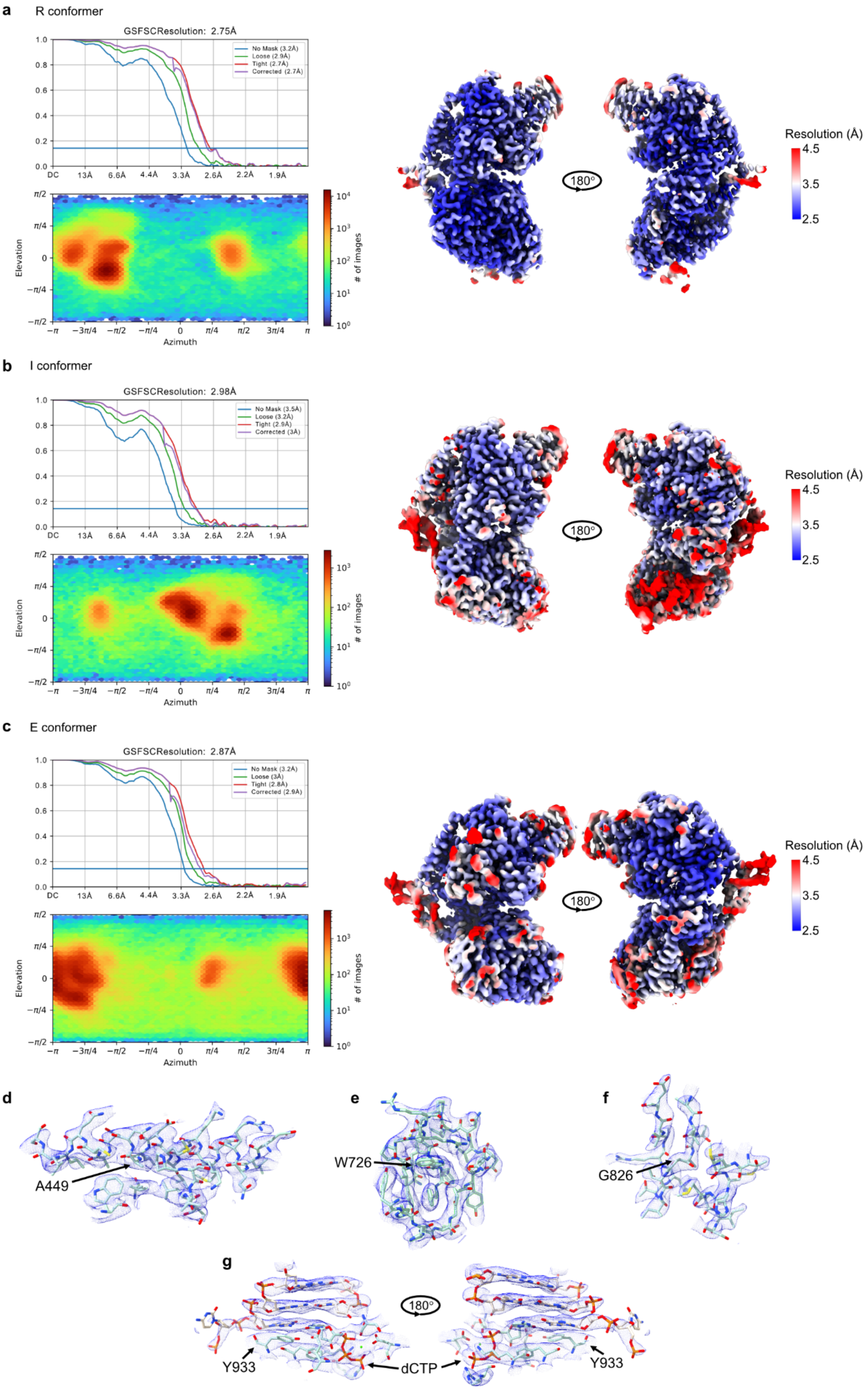
a
R conformer
GSFSCResolution: 2.75Å
No Mask (3.2Å)
Loose (2.9Å)
Tight (2.7Å)
Corrected (2.7Å)
Elevation
Azimuth
# of images
180°
Resolution (Å)
4.5
3.5
2.5
b
I conformer
GSFSCResolution: 2.98Å
No Mask (3.5Å)
Loose (3.2Å)
Tight (2.9Å)
Corrected (3Å)
Elevation
Azimuth
# of images
180°
Resolution (Å)
4.5
3.5
2.5
c
E conformer
GSFSCResolution: 2.87Å
No Mask (3.2Å)
Loose (3Å)
Tight (2.8Å)
Corrected (2.9Å)
Elevation
Azimuth
# of images
180°
Resolution (Å)
4.5
3.5
2.5
d
A449
e
W726
f
G826
g
180°
Y933
dCTP
Y933

Supplementary Fig. 4. Assessment of the cryo-EM maps for mPolγ.

FSC resolution (FSC threshold = 0.143) determined during the final non-uniform refinement in cryoSPARC, the orientation distribution plot and local resolution estimation (FSC threshold = 0.5) for; **a**, R conformer; **b**, I conformer; **c**, E conformer.

d–g. The model of the mouse R conformer structure at the sites of residues of interest, with map densities displayed as mesh.

d. A449 region. The map is contoured at a level of 6.2.

e. W726 region. The map is contoured at a level of 3.0.

f. G826 region. The map is contoured at a level of 10.0.

g. Y933 and active site region. The map is contoured at a level of 7.0.

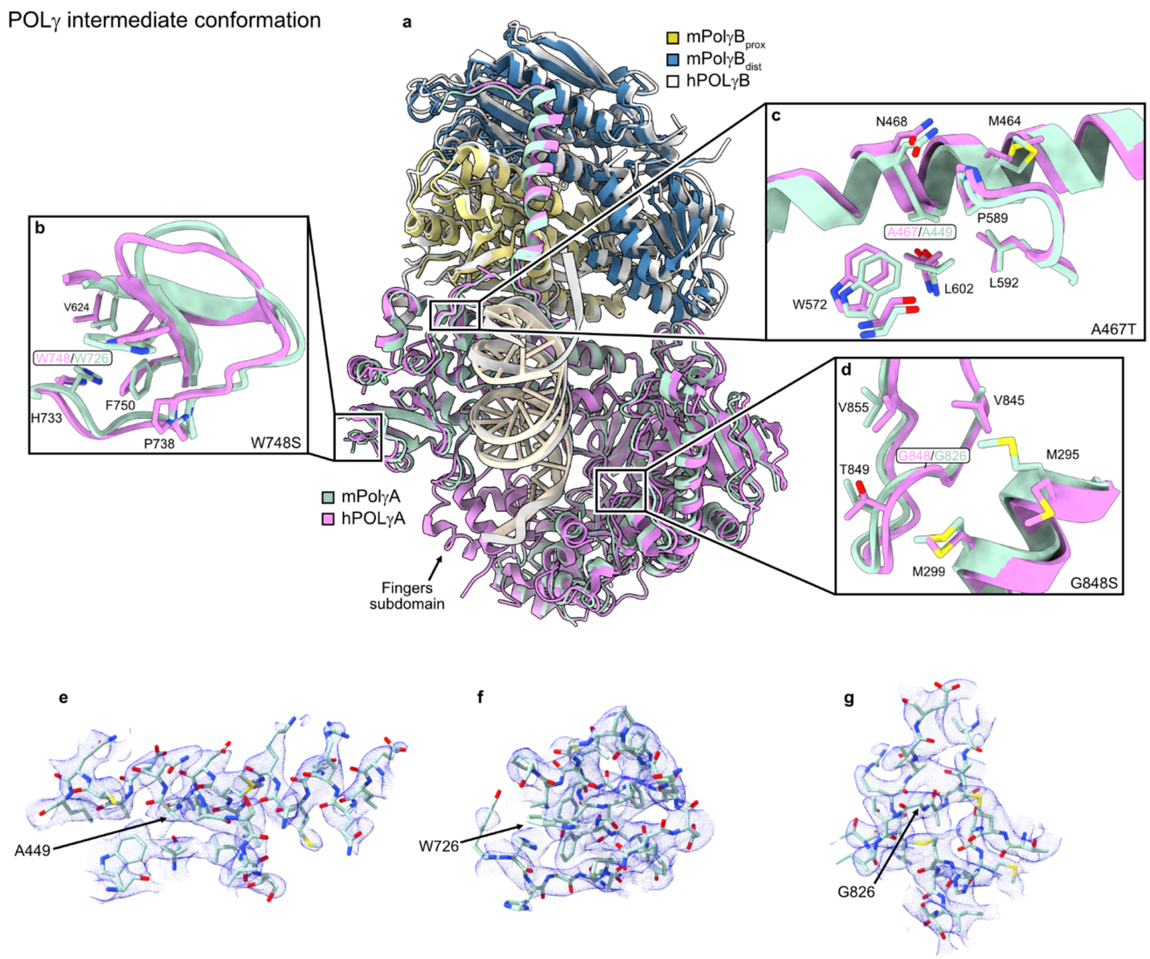

Supplementary Fig. 5. Structural comparison of the hPOLγ and mPolγ intermediate conformers.

a. Overview of overlaid hPOLγ (PDB ID: 8D3R) and mPolγ intermediate conformers. The position of the hPOLγA (magenta) and mPolγA (green) subunits are near identical, which also applies for the position of the DNA (grey and beige for human and mouse structures, respectively). In the POLγB region, there is a small shift between in the distal mPolγB (blue) and distal hPOLγB (white), while the proximal mPolγB (yellow) is still aligned to the proximal hPOLγB (white).

b. Closeup of the A467/A449 position and surrounding residues.

c. Closeup of the W748/W726 position and surrounding residues.

d. Closeup of the G848/G826 position and surrounding residues. In panels b–d, the surrounding residues are numbered according to the human sequence.

e–g. The model of the mouse I conformer structure at the sites of residues of interest, with map densities displayed as mesh.

e. A449 region. The map is contoured at a level of 6.5.

f. W726 region. The map is contoured at a level of 3.5.

g. G826 region. The map is contoured at a level of 6.0.

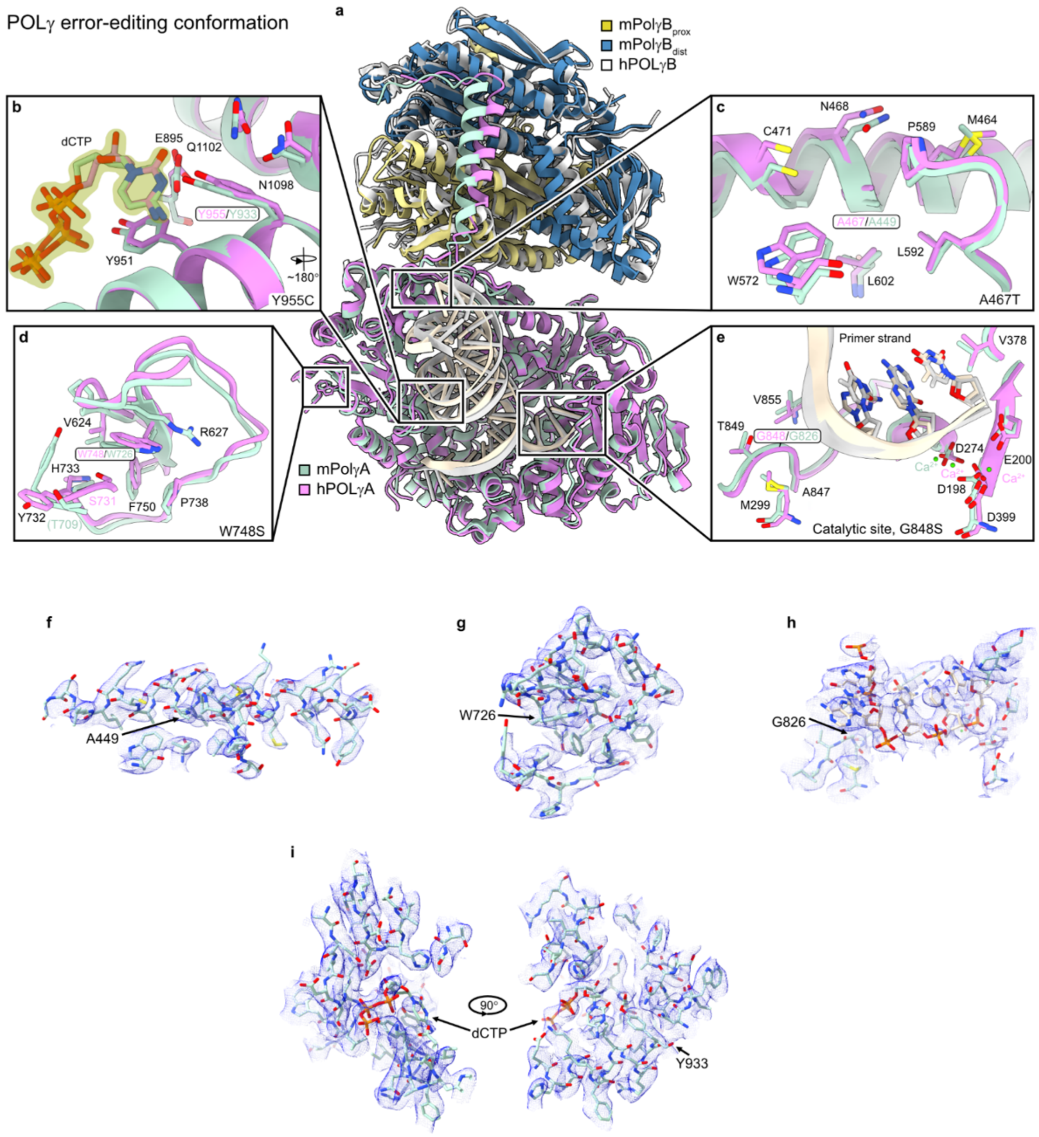

Supplementary Fig. 6. Structural comparison of the hPOLγ and mPolγ error-editing conformers.

a. Overview of overlaid hPOLγ (PDB ID: 8D42) and mPolγ error-editing conformers. The position of the hPOLγA (magenta) and mPolγA (green) subunits are near identical, which also applies for the position of the DNA (grey and beige for human and mouse structures, respectively). However, the L-helix and POLγB are in different positions, where hPOLγB (white) is shifted compared to both the distal mPolγB (blue) and proximal mPolγB (yellow).

b. Closeup of the Y955/Y933 residues and the surrounding residues. The incoming nucleotide (dCTP) is highlighted in yellow.

c. Closeup of the A467/A449 position and surrounding residues. The main difference is that another rotamer has been used for Y732 sidechain in the human model, however, while

reviewing of the published map (EMD-27172) of the human version, it is unclear which rotamer is the correct one. The discrepancy may therefore be a modeling error.

d. Closeup of the W748/W726 position and surrounding residues.

e. Closeup of the catalytic site (EXO) and G848/G826 position and surrounding residues. In panels **b–e**, the surrounding residues are numbered according to the human sequence.

f–i. The model of the mouse E conformer structure at the sites of residues of interest, with map densities displayed as mesh.

f. A449 region. The map is contoured at a level of 10.2.

g. W726 region. The map is contoured at a level of 3.4.

h. G826 region and exonuclease site. The map is contoured at a level of 2.8.

i. Y933 and active site. The map is contoured at a level of 3.5.

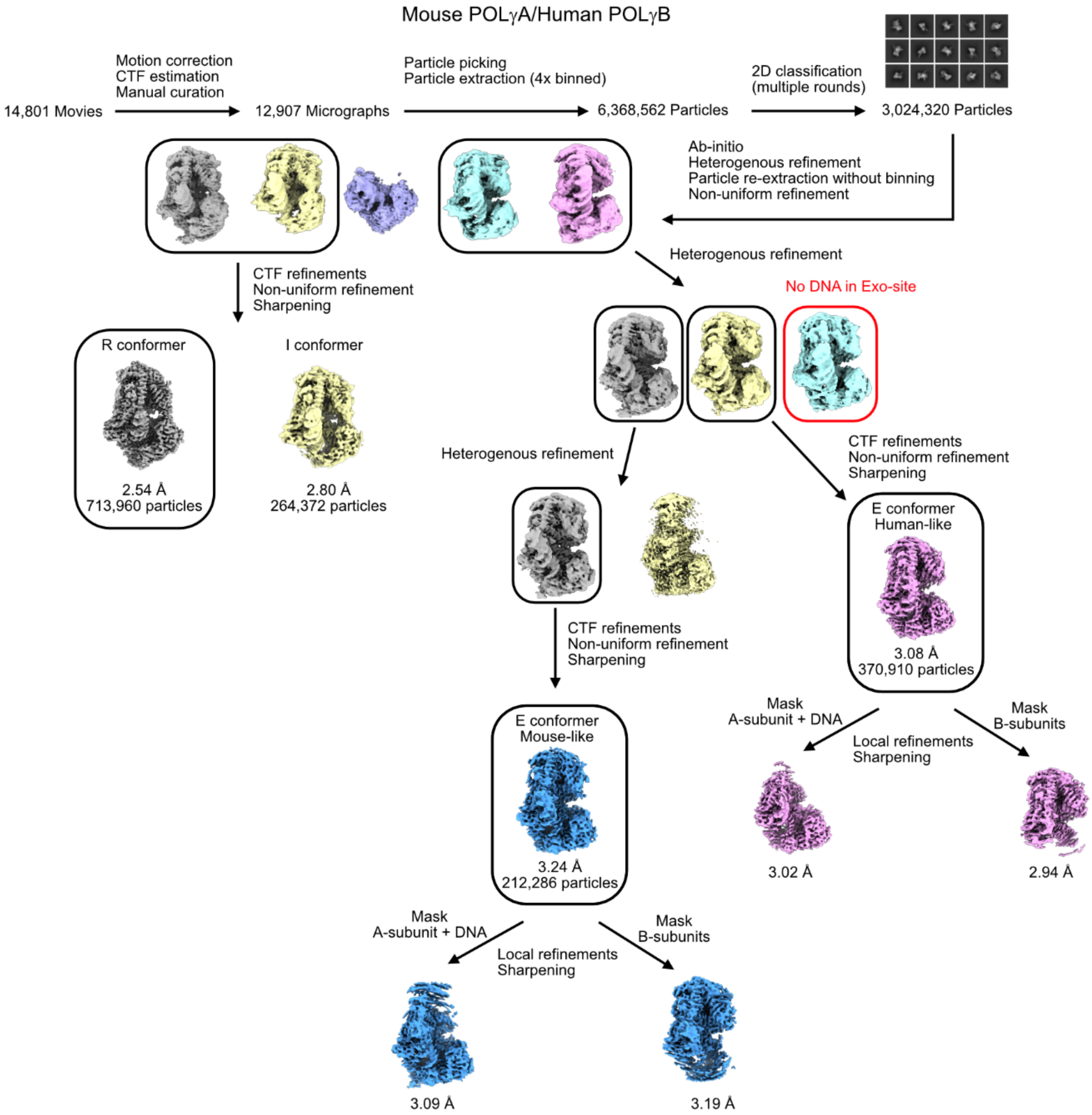

Supplementary Fig. 7. Cryo-EM processing workflow for the chimeric mPolγA/hPOLγB complex.

The data were imported into cryoSPARC, which was used for processing and generating the 3D volumes. Both R and I conformers were observed as well as two E conformers; one mouse-like and one human-like. The R conformer and the two E conformers were used for modelling.

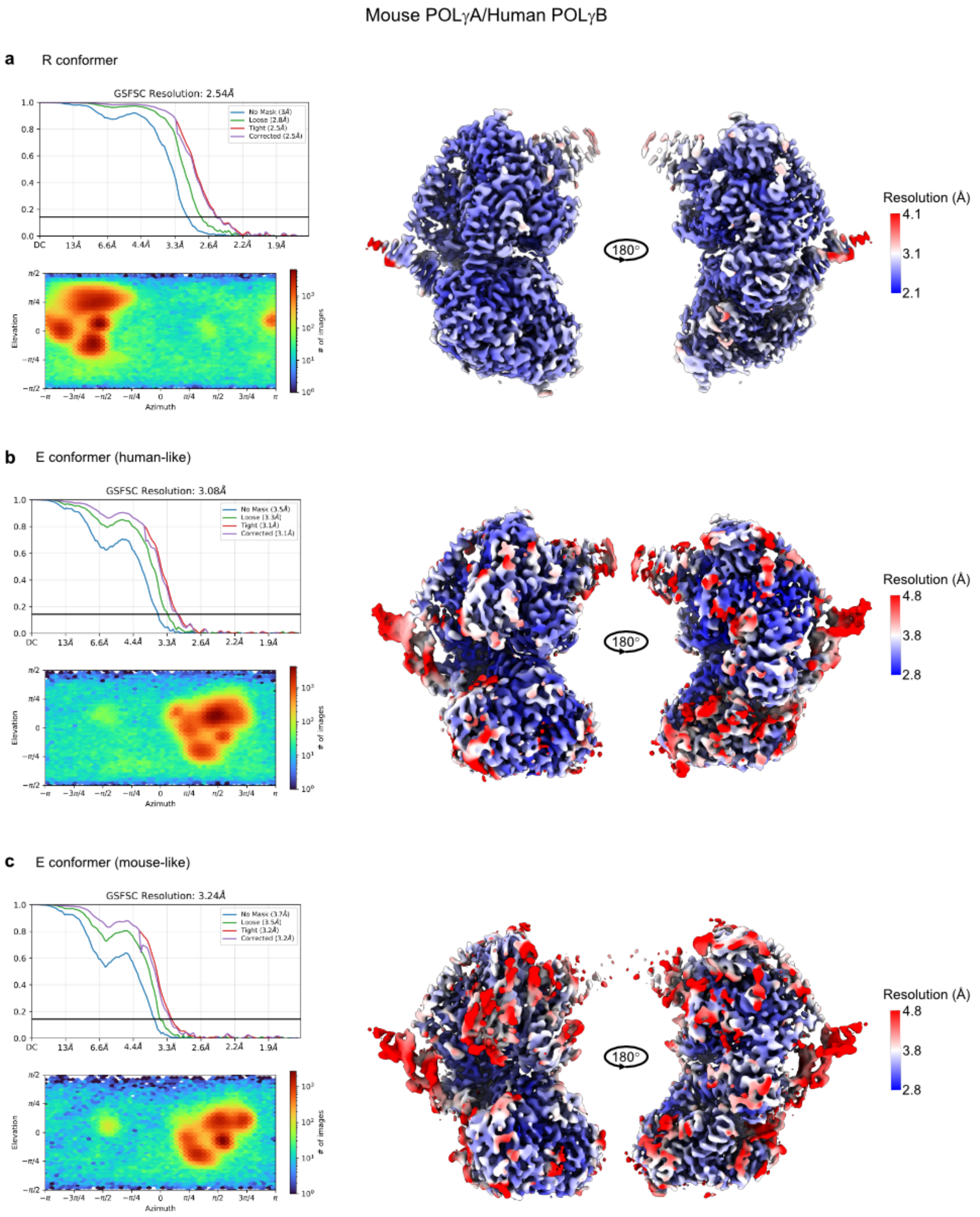

Supplementary Fig. 8. Assessment of the cryo-EM maps for the chimeric mPolγA/hPOLγB complex.

FSC resolution (FSC threshold = 0.143) determined during the final non-uniform refinement in cryoSPARC, the orientation distribution plot and local resolution estimation (FSC threshold = 0.5) for; **a**, R conformer; **b**, E conformer (human-like); **c**, E conformer (mouse-like).

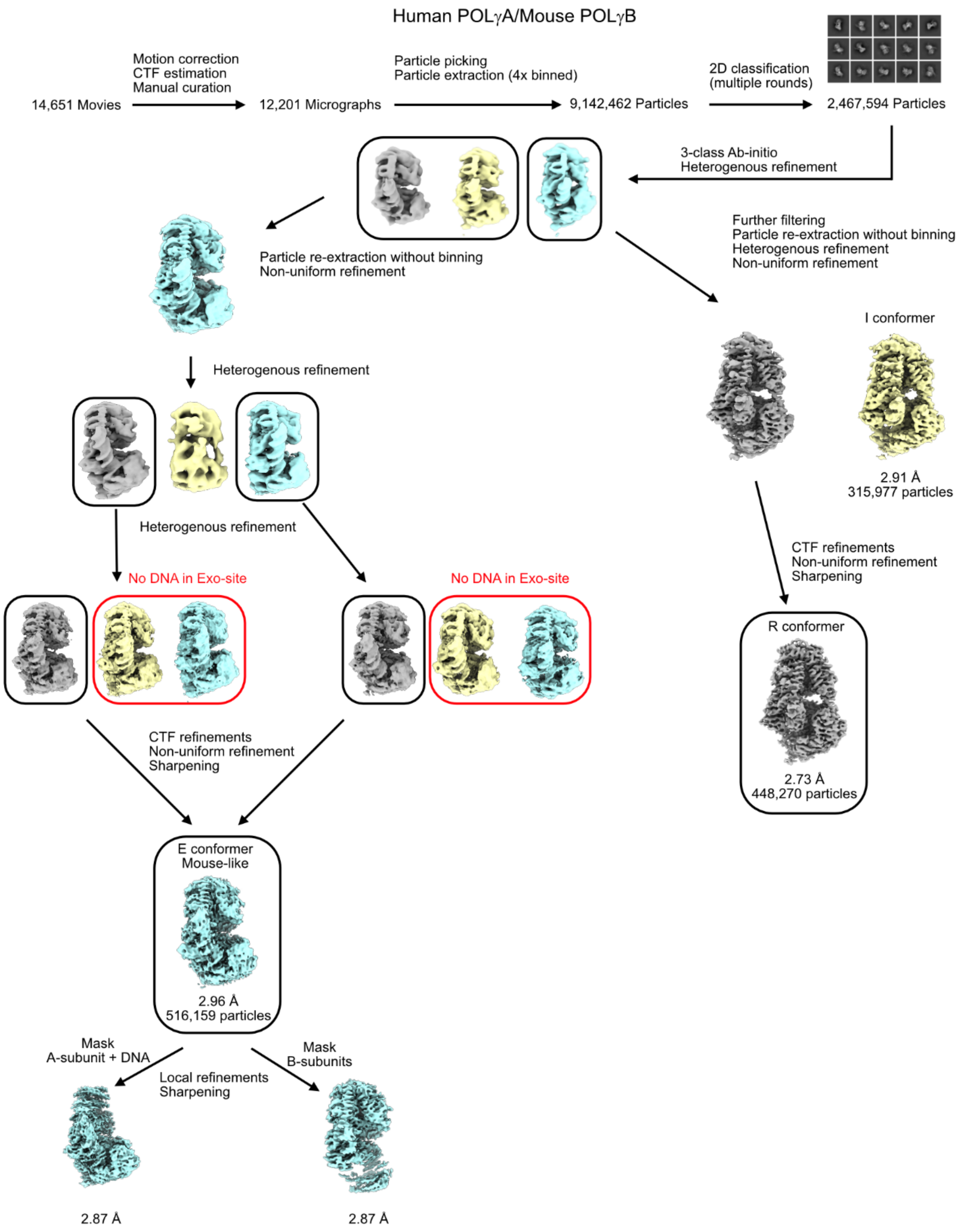

Supplementary Fig. 9. Cryo-EM processing workflow for the chimeric hPOLγA/mPolγB complex.

The data were imported into cryoSPARC, which was used for processing and generating the 3D volumes. Both R and I conformers were observed as well as one E conformer (mouse-like). The R conformer and the E conformers were used for modelling.

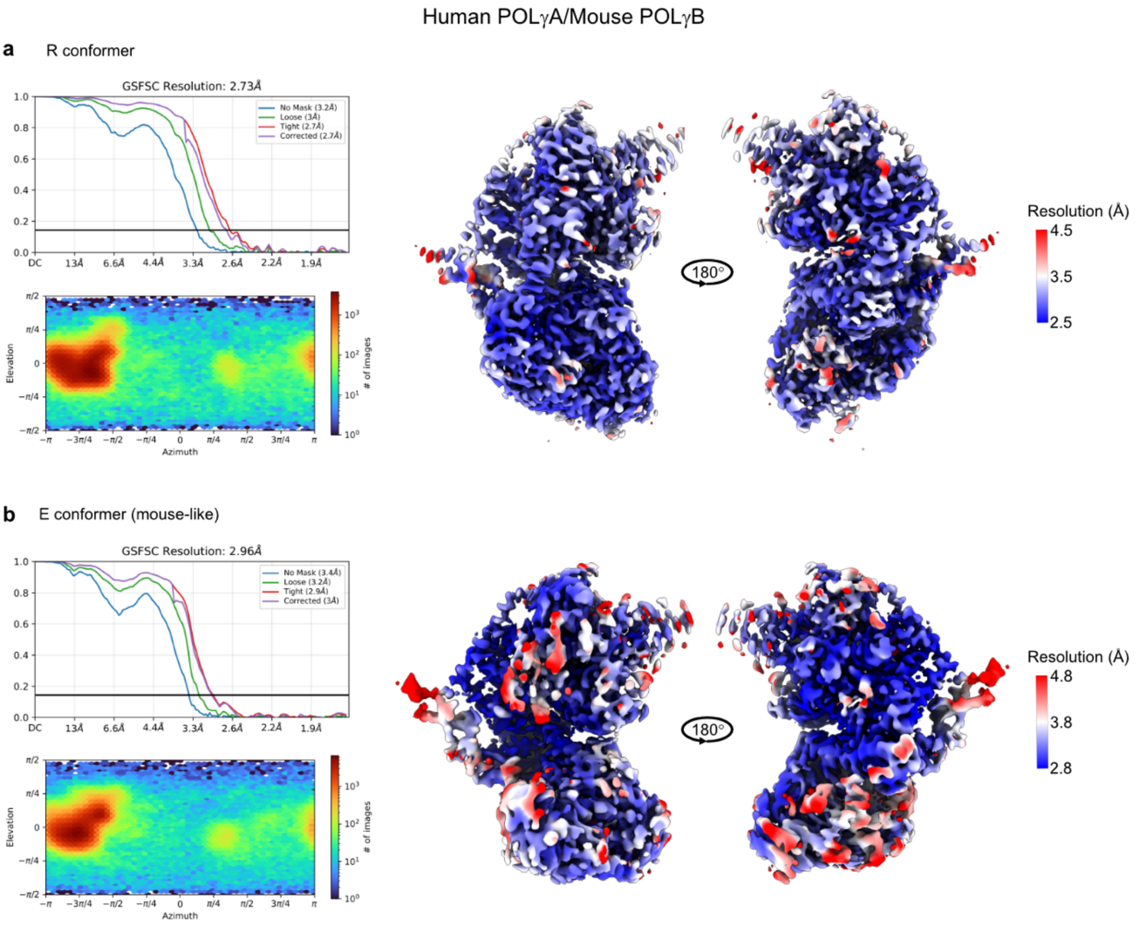

Supplementary Fig. 10. Assessment of the cryo-EM maps for the chimeric hPOLγA/mPolγB complex.

FSC resolution (FSC threshold = 0.143) determined during the final non-uniform refinement in cryoSPARC, the orientation distribution plot and local resolution estimation (FSC threshold = 0.5) for; **a**, R conformer; **b**, E conformer (mouse-like).

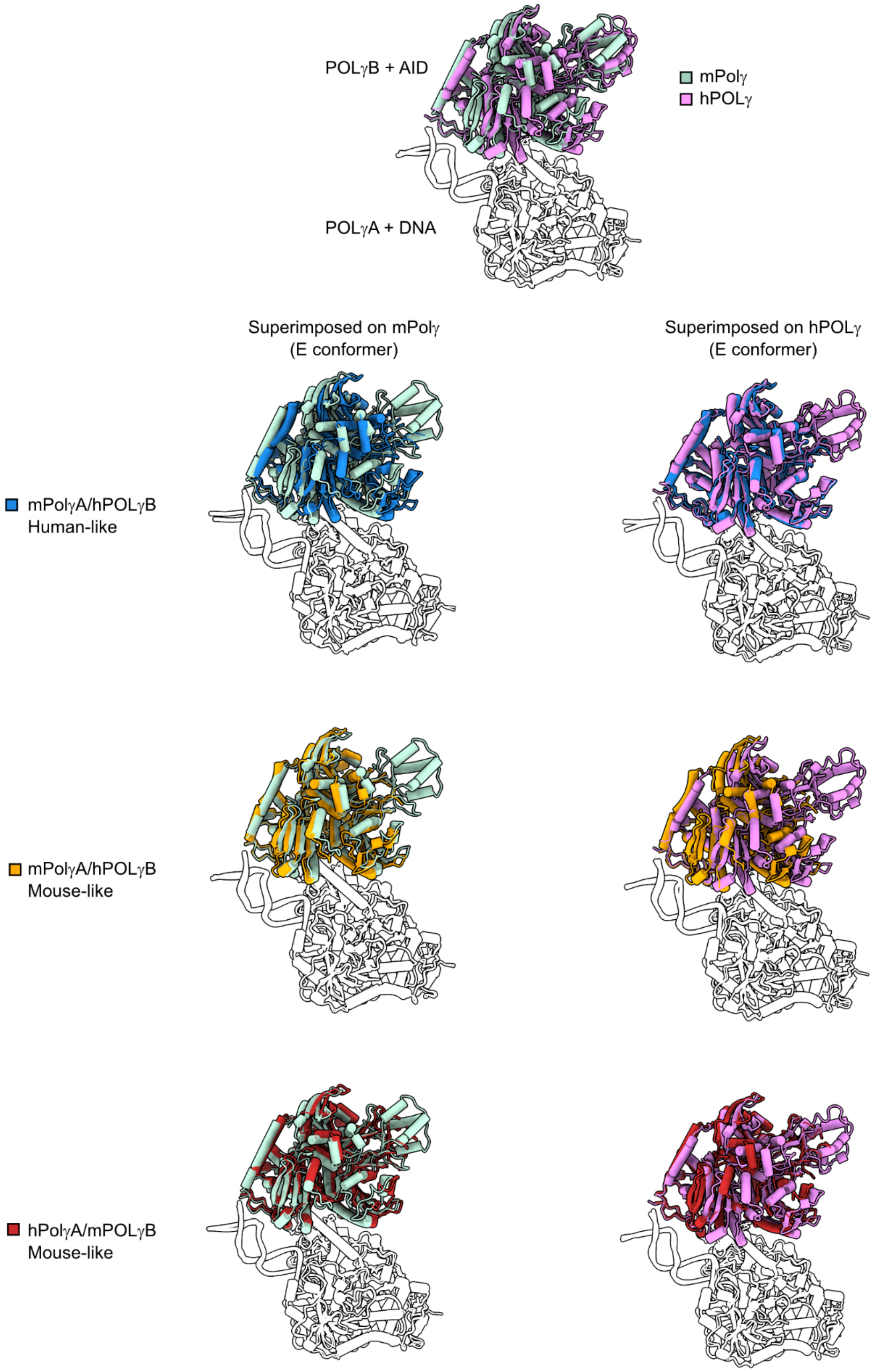
POLγB + AID
POLγA + DNA
mPolγ
hPOLγ
Superimposed on mPolγ
(E conformer)
Superimposed on hPOLγ
(E conformer)
mPolγA/hPOLγB
Human-like
mPolγA/hPOLγB
Mouse-like
hPolγA/mPOLγB
Mouse-like

Supplementary Fig. 11. Comparison between mPolγ or hPOLγ and chimeric POLγ complexes.

The E conformers of chimeric POLγ (mAhB or hAmB) were superimposed on mPolγ or hPOLγ for comparison. POLγA and DNA are outlined while POLγB and the AID are colored. There are only small differences in the catalytic subunit, however, the position of the POLγB subunits vary. In the mAhB chimera, both human-like (blue) and mouse-like (orange) conformations can be observed. In the hAmB chimera, only the mouse-like (red) conformation is observed. For hAmB chimera, the DNA in the exo-site were relatively poor compared to the mAhB variants, indicating more flexibility or less stability in this conformation.

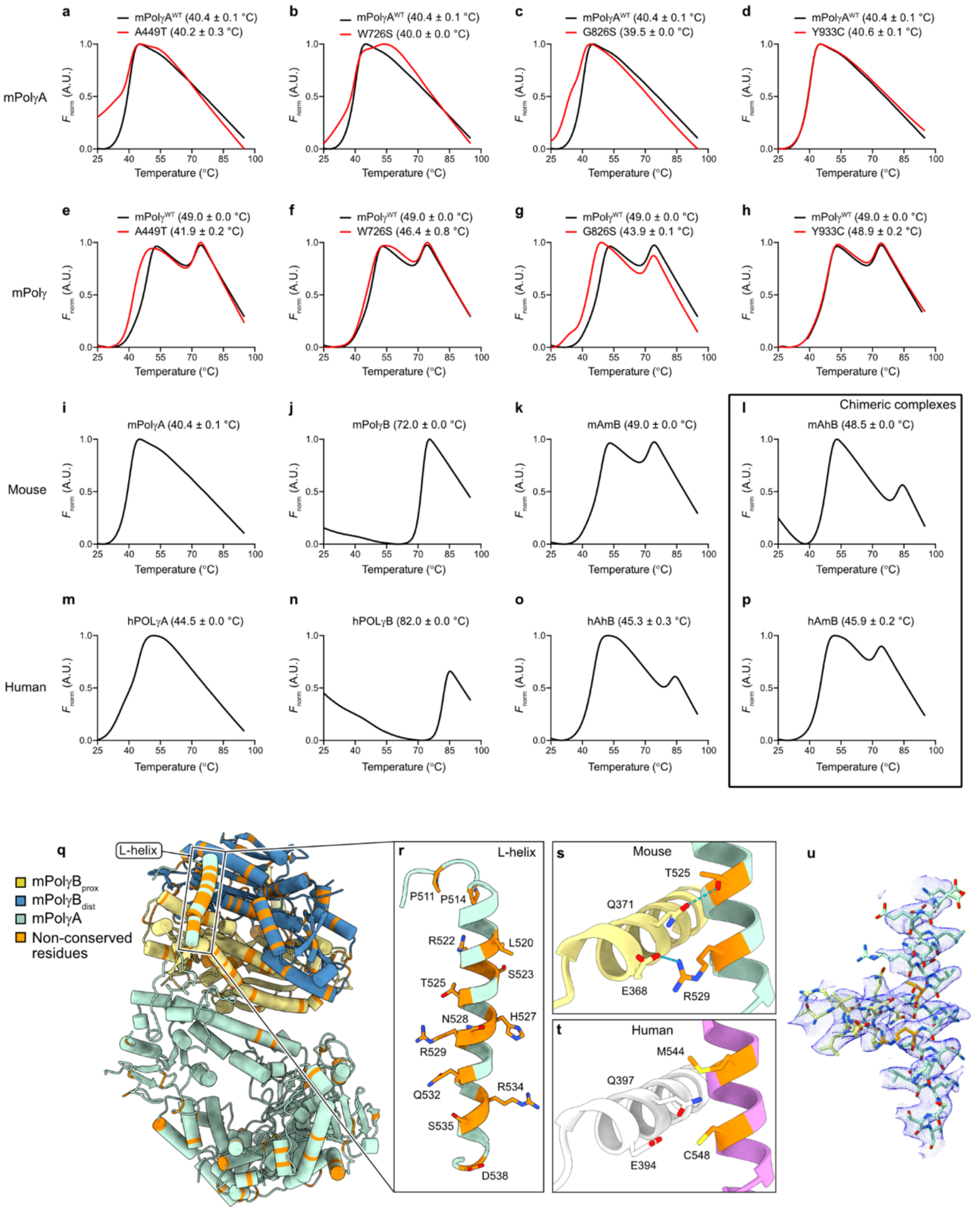

Supplementary Fig. 12. Thermal stability of mPolγ^{WT} and mutants

Representative plots of differential scanning fluorimetry. Values are presented as normalized fluorescence (F_{norm}) in arbitrary units (A.U.). The melting temperature (T_m) is given as mean ± s.d., n = 3 independent experiments for PolγA in all panels except in **j** and **n** where T_m is given for PolγB.

a–d. Melting curves for mPolγA^{A449T}, mPolγA^{W726S}, mPolγA^{G826S}, mPolγA^{Y933C} and mPolγA^{WT}.

e–h. Melting curves for mPolγA^{A449T}, mPolγA^{W726S}, mPolγA^{G826S}, mPolγA^{Y933C} and mPolγA^{WT} in complex with mPolγB.

i–l. Melting curves for mPolγA^{WT}, mPolγB^{WT}, mPolγ^{WT} and the chimeric mPolγA^{WT}/hPOLγB^{WT} (mAhB) complex. The melting curve in **i** was also used in panels **a–d**. The melting curve in **k** was also used in panels **e–h.**

m–p. Melting curves for hPOLγA^{WT}, hPOLγB^{WT}, hPOLγ^{WT} and the chimeric hPOLγA^{WT}/mPolγB^{WT} (hAmB) complex.

q. Adapted version of Fig 4a of the mPolγ E conformer (DNA omitted). Non-conserved residues between human and mouse are shown in orange in both POLγA and POLγB. Many non-conserved residues are clustered in POLγB and the L-helix.

r. Closeup of the L-helix with residues displayed as sticks.

s–t. The mPolγA L-helix can form electrostatic interactions with the proximal mPolγB subunit via the T525 and R529 residues. However, in the hPOLγA L-helix, these residues are substituted to M544 and C548, which lacks the ability to form strong interactions with the proximal hPOLγB subunit.

u. The helices in **s** displayed as sticks with the map density displayed as mesh. The map is contoured at a level of 4.0.

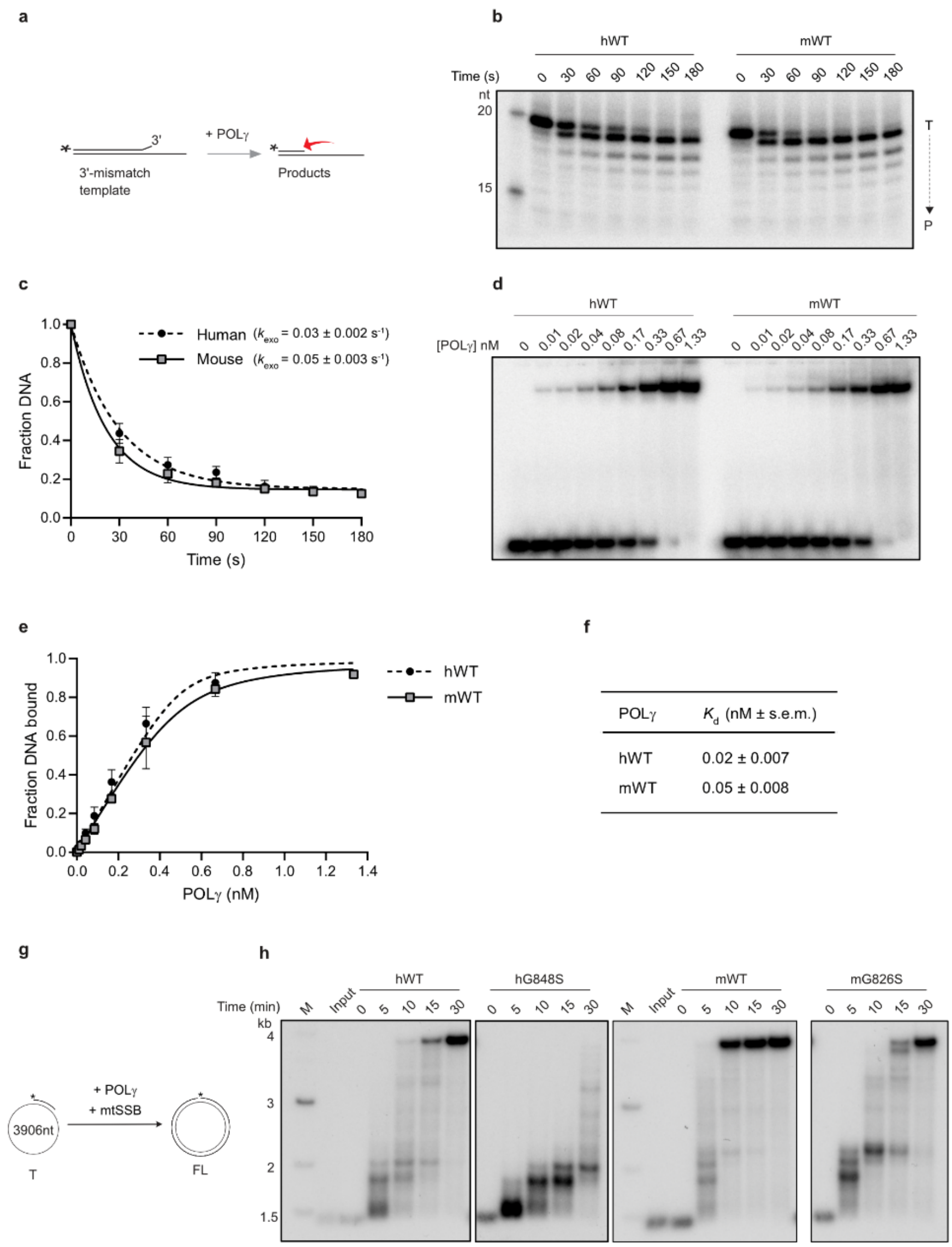

Supplementary Fig. 13. Comparable exonuclease activity between human and mouse Polγ holoenzymes.

a. Schematic representation of the exonuclease assay used to investigate the effects of various mutant POLγ proteins on proofreading activity using a 3'-mismatch template.

b. Time-course experiments reveal that hPOLγ^{WT} and mPolγ^{WT} efficiently remove a mismatch at the 3′-end. The reactions were incubated with the mismatch-template at 37 °C in the absence of dNTPs and for the indicated times. The products were separated on 10% urea-PAGE

sequencing gels. The positions of products (P) and the radioactively labeled mismatch template (T) are indicated. $n = 3$ independent experiments.

c. The fraction non-degraded 20-mer was quantified and plotted versus time to visualize the DNA degradation. Data are presented as the mean ± s.d., $n = 3$ independent experiments. The data was fitted to an exponential decay equation to determine the rate constants (k_{exo}) for human and mouse. Values are given with errors in s.e.m.

d. The DNA binding activity of a stalled elongation complex of hPOLγ^{WT} and mPolγ^{WT} was analyzed by EMSA using a primed DNA template. Source data are provided with this paper.

e. The binding ratio was plotted against polymerase complex concentration. Data are presented as the mean ± s.d., $n = 2$ independent experiments, and the binding curves were fitted to a non-linear quadratic equation to determine the dissociation constants, K_d.

f. Summary of the determined K_d values, with errors given as s.e.m.

g-h Time-course experiments, performed as outlined in Fig. 1a but with a single-stranded DNA template of 3900 nt instead of 7249 nt, reveal that the replication rate is higher with the mPolγ variants than with the hPOLγ variants. POLγ and mtSSB were incubated with a primed, single-stranded DNA template and 10 µM dNTP at 37 °C for the indicated times and analyzed on a 0.8% native agarose gel. The positions of the full-length (FL) product and the radioactively labeled primer template (T) are indicated. $n = 2$ independent experiments.

Source data are provided as a Source Data file.

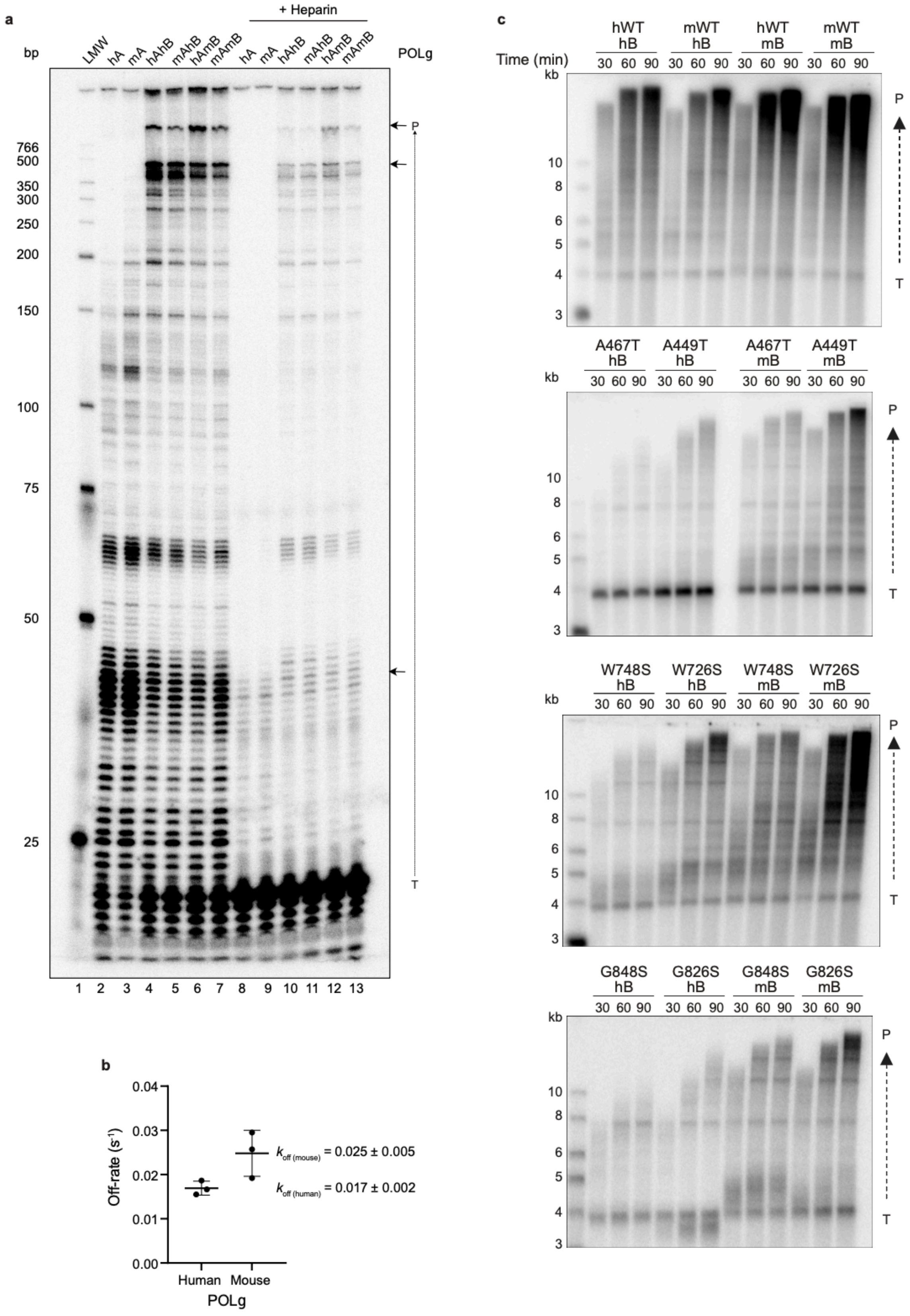

a
+ Heparin
bp
LMW
hA
mA
hAhB
mAhB
hAmB
mAmB
hA
mA
hAhB
mAhB
hAmB
mAmB
POLg
766
500
350
300
250
200
150
100
75
50
25
P
T
1 2 3 4 5 6 7 8 9 10 11 12 13
b
0.04
0.03
0.02
0.01
0.00
Off-rate (s-1)
$k_{off\ (mouse)}$ = 0.025 ± 0.005
$k_{off\ (human)}$ = 0.017 ± 0.002
Human
Mouse
POLg
c
hWT hB
mWT hB
hWT mB
mWT mB
Time (min)
30 60 90 30 60 90 30 60 90 30 60 90
kb
10
8
6
5
4
3
P
T
A467T hB
A449T hB
A467T mB
A449T mB
30 60 90 30 60 90 30 60 90 30 60 90
W748S hB
W726S hB
W748S mB
W726S mB
30 60 90 30 60 90 30 60 90 30 60 90
G848S hB
G826S hB
G848S mB
G826S mB
30 60 90 30 60 90 30 60 90 30 60 90

Supplementary Fig. 14. Comparable processivity of human and mouse polγ holoenzymes.
a. To measure processivity, the indicated POLγ variants were pre-incubated on ice with a primed, single-stranded DNA template. DNA synthesis was initiated by adding either dNTP alone or dNTP and heparin, and the reactions were allowed to proceed for 5 minutes at 37 °C. The products were separated on 8% urea-PAGE sequencing gels. The positions of the products (P) and the radioactively labeled template (T) are indicated. In the absence of heparin, the proteins can rebind to continue DNA synthesis. Heparin is used to trap free POLγ, preventing the enzyme from rebinding and continuing DNA synthesis after dissociation from the template. Arrows indicate the longest products identified for POLγA, both in isolation and with the holoenzymes. *n* = 2 independent experiments.
b. The dissociation rate constants, k_{off} (s^{-1}) determined for hPOLγ and mPolγ are shown. Off-rate was determined using a single-nucleotide incorporation assay at varying time points. The fraction of 26-mer product formed was plotted against time (s). Following linear regression fitting, k_{off} was calculated by dividing the slope of the linear phase by the concentration of POLγ in the reaction. Data are presented as mean ± s.d., *n* = 2 independent experiments.
c. Time-course experiments performed as outlined in Fig. 2a reveal that mPolγB stimulates replication better than hPOLγB for both hPOLγA^{WT} and mPolγA^{WT}, as well as for all mutant mouse and human variants during TWINKLE-dependent rolling-circle DNA replication. mPolγ^{WT} and hPOLγ^{WT}, mutant variants and mixing variants, as indicated, were incubated with TWINKLE, mtSSB, and the rolling-circle template at 37 °C for the indicated times. The polymerase concentration is always the same; when mixing two variants, half of each is used to match the final concentration in single-polymerase reactions. DNA synthesis was monitored as the incorporation of radioactive nucleotides, and the products of increasing length were separated on a 0.8% alkaline agarose gel. Template (T); Products (P). *n* = 2 independent experiments.
Source data are provided as a Source Data file.

a

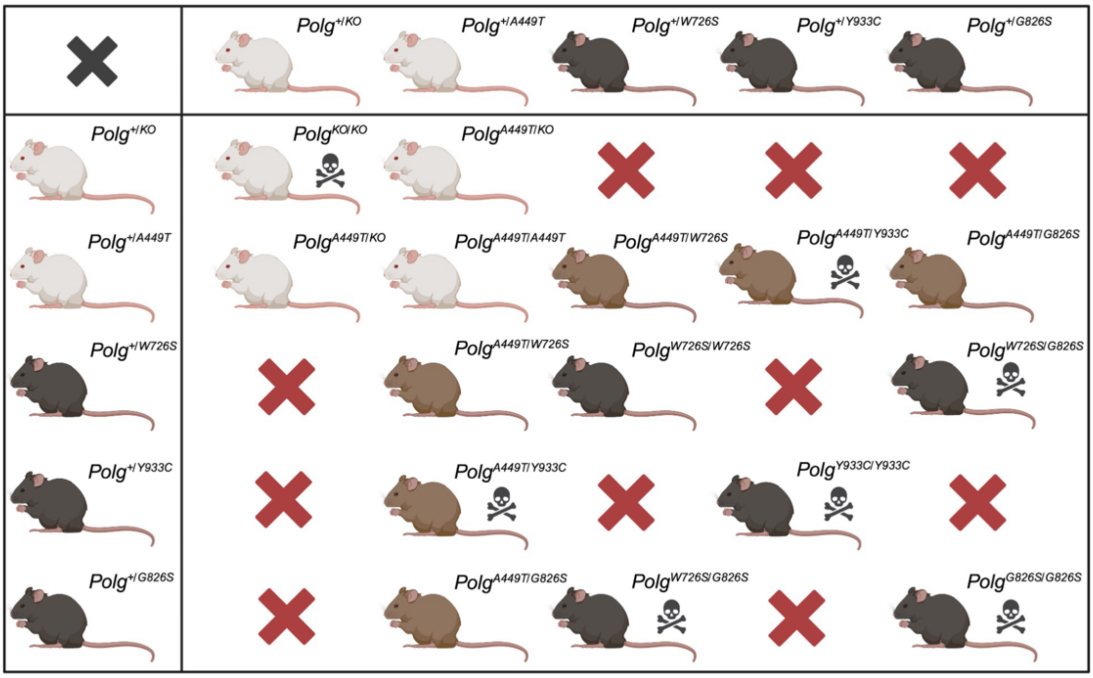
Polg+/KO
Polg+/A449T
Polg+/W726S
Polg+/Y933C
Polg+/G826S
PolgKO/KO
PolgA449T/KO
PolgA449T/A449T
PolgA449T/W726S
PolgA449T/Y933C
PolgA449T/G826S
PolgW726S/W726S
PolgW726S/G826S
PolgY933C/Y933C
PolgG826S/G826S

b

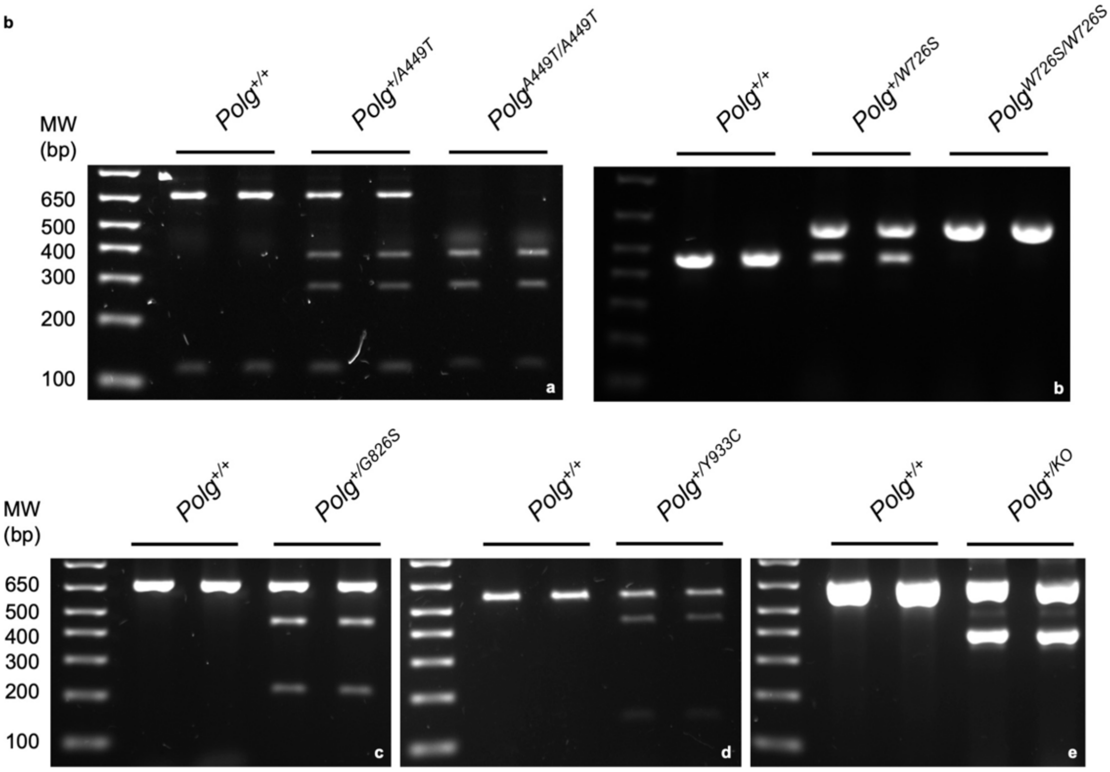
MW (bp)
650
500
400
300
200
100
Polg+/+
Polg+/A449T
PolgA449T/A449T
Polg+/W726S
PolgW726S/W726S
Polg+/G826S
Polg+/Y933C
Polg+/KO
a
b
c
d
e

Supplementary Fig. 15. Breeding scheme and genotyping.

a. Red crosses indicate matings that have not been done; skulls and crossbones indicate pre-weaning lethality. Created in BioRender. Zuppardo, A. (2025) https://BioRender.com/difmonx.

b. Genotyping PCR of wild-type and mutant mice. *a*: $Polg^{A449T}$ (+/+: 652bp and117 bp; +/-: 652bp, 372bp, 280bp and 117bp; -/-: 372bp, 280bp and 117bp). *b*: $Polg^{W726S}$ (+/+: 460bp; +/-: 460bp and 580bp; -/-: 580bp). *c*: $Polg^{G826S}$ (+/+: 667bp; +/-: 459bp and 208bp). *d*: $Polg^{Y933C}$ (+/+: 561bp; +/-: 131bp and 430bp). *e*: $Polg^{KO}$ (+/+: 664bp; +/-: 664bp and 399bp).

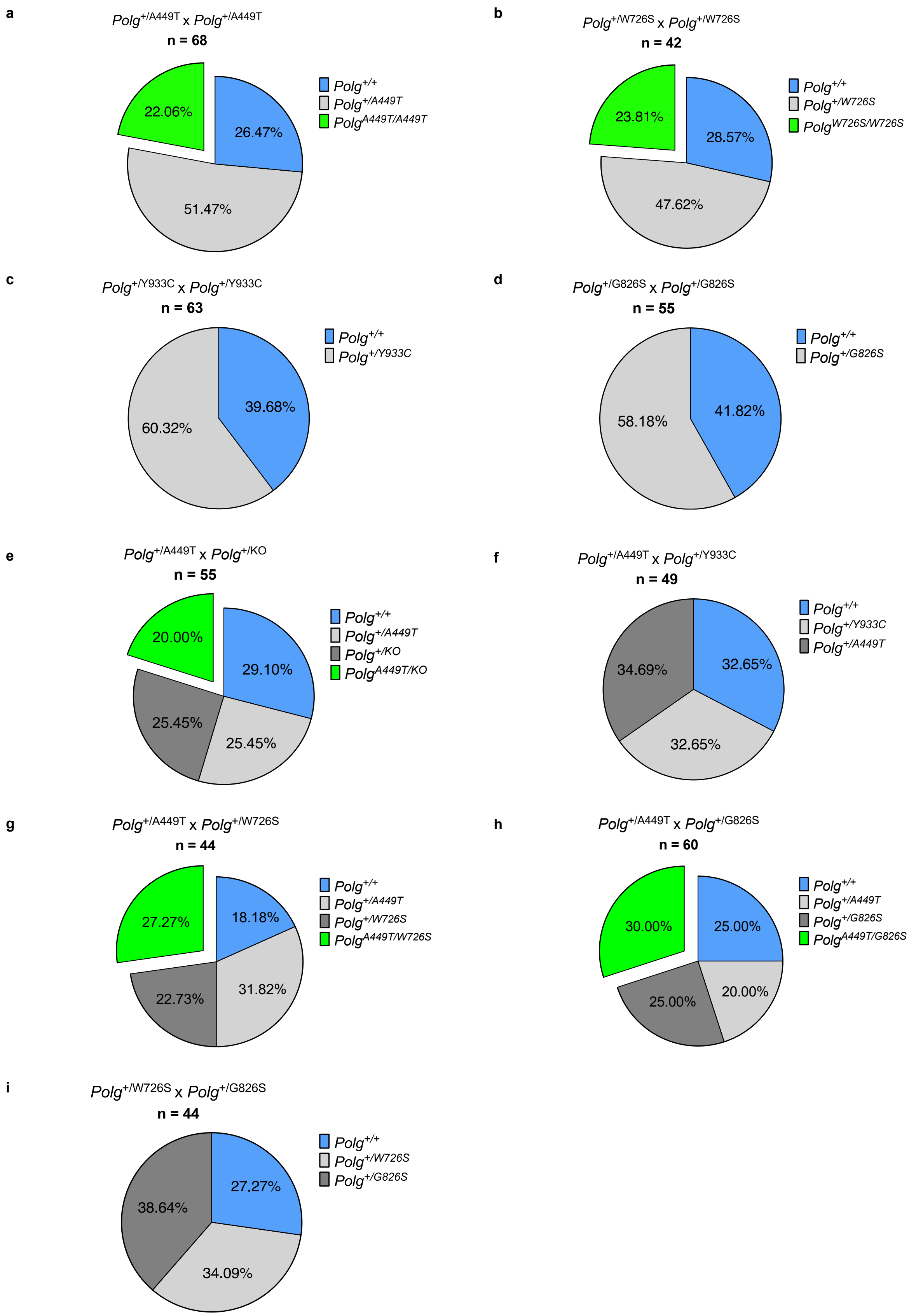
a
Polg$^{+/A449T}$ x *Polg*$^{+/A449T}$
n = 68
22.06%
26.47%
51.47%
Polg$^{+/+}$
Polg$^{+/A449T}$
Polg$^{A449T/A449T}$
b
Polg$^{+/W726S}$ x *Polg*$^{+/W726S}$
n = 42
23.81%
28.57%
47.62%
Polg$^{+/+}$
Polg$^{+/W726S}$
Polg$^{W726S/W726S}$
c
Polg$^{+/Y933C}$ x *Polg*$^{+/Y933C}$
n = 63
39.68%
60.32%
Polg$^{+/+}$
Polg$^{+/Y933C}$
d
Polg$^{+/G826S}$ x *Polg*$^{+/G826S}$
n = 55
41.82%
58.18%
Polg$^{+/+}$
Polg$^{+/G826S}$
e
Polg$^{+/A449T}$ x *Polg*$^{+/KO}$
n = 55
20.00%
29.10%
25.45%
25.45%
Polg$^{+/+}$
Polg$^{+/A449T}$
Polg$^{+/KO}$
Polg$^{A449T/KO}$
f
Polg$^{+/A449T}$ x *Polg*$^{+/Y933C}$
n = 49
34.69%
32.65%
32.65%
Polg$^{+/+}$
Polg$^{+/Y933C}$
Polg$^{+/A449T}$
g
Polg$^{+/A449T}$ x *Polg*$^{+/W726S}$
n = 44
27.27%
18.18%
22.73%
31.82%
Polg$^{+/+}$
Polg$^{+/A449T}$
Polg$^{+/W726S}$
Polg$^{A449T/W726S}$
h
Polg$^{+/A449T}$ x *Polg*$^{+/G826S}$
n = 60
30.00%
25.00%
25.00%
20.00%
Polg$^{+/+}$
Polg$^{+/A449T}$
Polg$^{+/G826S}$
Polg$^{A449T/G826S}$
i
Polg$^{+/W726S}$ x *Polg*$^{+/G826S}$
n = 44
27.27%
38.64%
34.09%
Polg$^{+/+}$
Polg$^{+/W726S}$
Polg$^{+/G826S}$

Supplementary Fig. 16. Mendelian distribution of the mutants.

Genotype frequencies of:

a. *Polg*$^{+/A449T}$ intercross (χ-square = 0.3235 with 2 df, ns p=0.8506),

b. *Polg*$^{+/W726S}$ intercross (χ-square = 0.2857 with 2 df, ns p=0.8669),

c. *Polg*$^{+/Y933C}$ intercross (χ-square = 22.52 with 2 df, **** p<0.0001),

d. *Polg*$^{+/\mathrm{G826S}}$ intercross (χ-square = 20.71 with 2 df, **** p<0.0001),

e. *Polg*$^{+/\mathrm{A449T}}$ x *Polg*$^{+/KO}$ (χ-square = 0.9273 with 3 df, ns p=0.8188),

f. *Polg*$^{+/\mathrm{A449T}}$ x *Polg*$^{+/Y933C}$ (χ-square = 16.39 with 3 df, *** p=0.0009),

g. *Polg*$^{+/\mathrm{A449T}}$ x *Polg*$^{+/W726S}$ (χ-square = 1.818 with 3 df, ns p=0.6110),

h. *Polg*$^{+/\mathrm{A449T}}$ x *Polg*$^{+/\mathrm{G826S}}$ (χ-square = 1.2 with 3 df, ns p=0.7530), and

i. *Polg*$^{+/W726S}$ x *Polg*$^{+/\mathrm{G826S}}$ (χ-square = 15.82 with 3 df, ** p=0.0012) mice.

The number of animals for every strain is reported above each graph. Source data are provided as a Source Data file

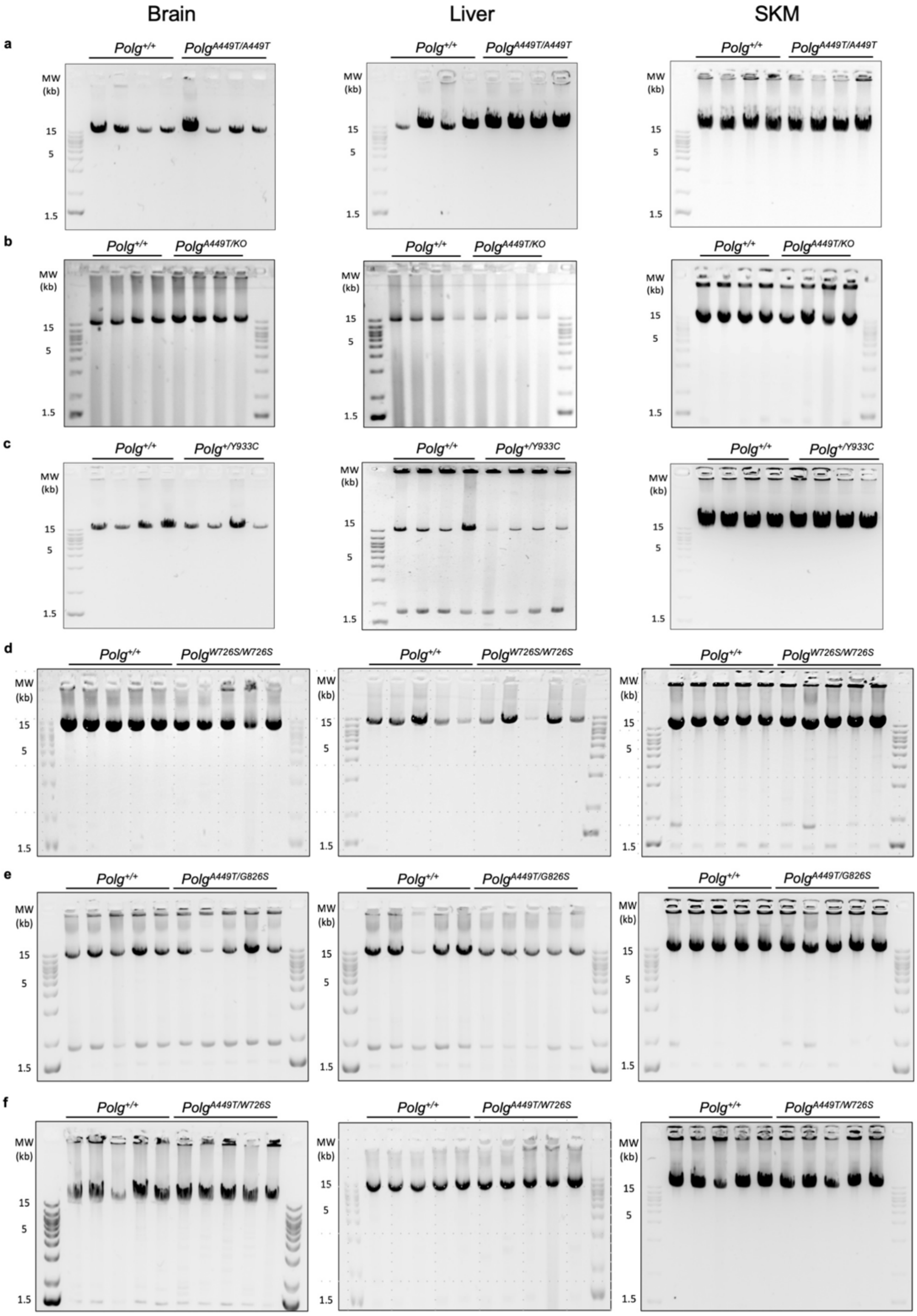

Brain
Liver
SKM
a
b
c
d
e
f
Polg+/+
PolgA449T/A449T
PolgA449T/KO
Polg+/Y933C
PolgW726S/W726S
PolgA449T/G826S
PolgA449T/W726S
MW (kb)
15
5
1.5

Supplementary Fig. 17. Long-range PCR performed in DNA isolated from brain, liver, and quadriceps (SKM) of 3-month-old mice.

a. $Polg^{A449T/A449T}$ (n = 4/genotype)

b. $Polg^{A449T/KO}$ (n = 4/genotype)

c. $Polg^{+/Y933C}$ (n = 4/genotype)

d. $Polg^{W726S/W726S}$ (n = 5/genotype)

e. $Polg^{A449T/G826S}$ (n = 5/genotype)

f. $Polg^{A449T/W726S}$ (n = 5/genotype)

Primers amplified a fragment of 15781 bp of the mtDNA. An aspecific band at 1.8 Kb is present in both mutants and wild-type in some gels. The bands were visualized by SYBR™ safe staining. Each lane represents a biological replicate. Source data are provided as a Source Data file.

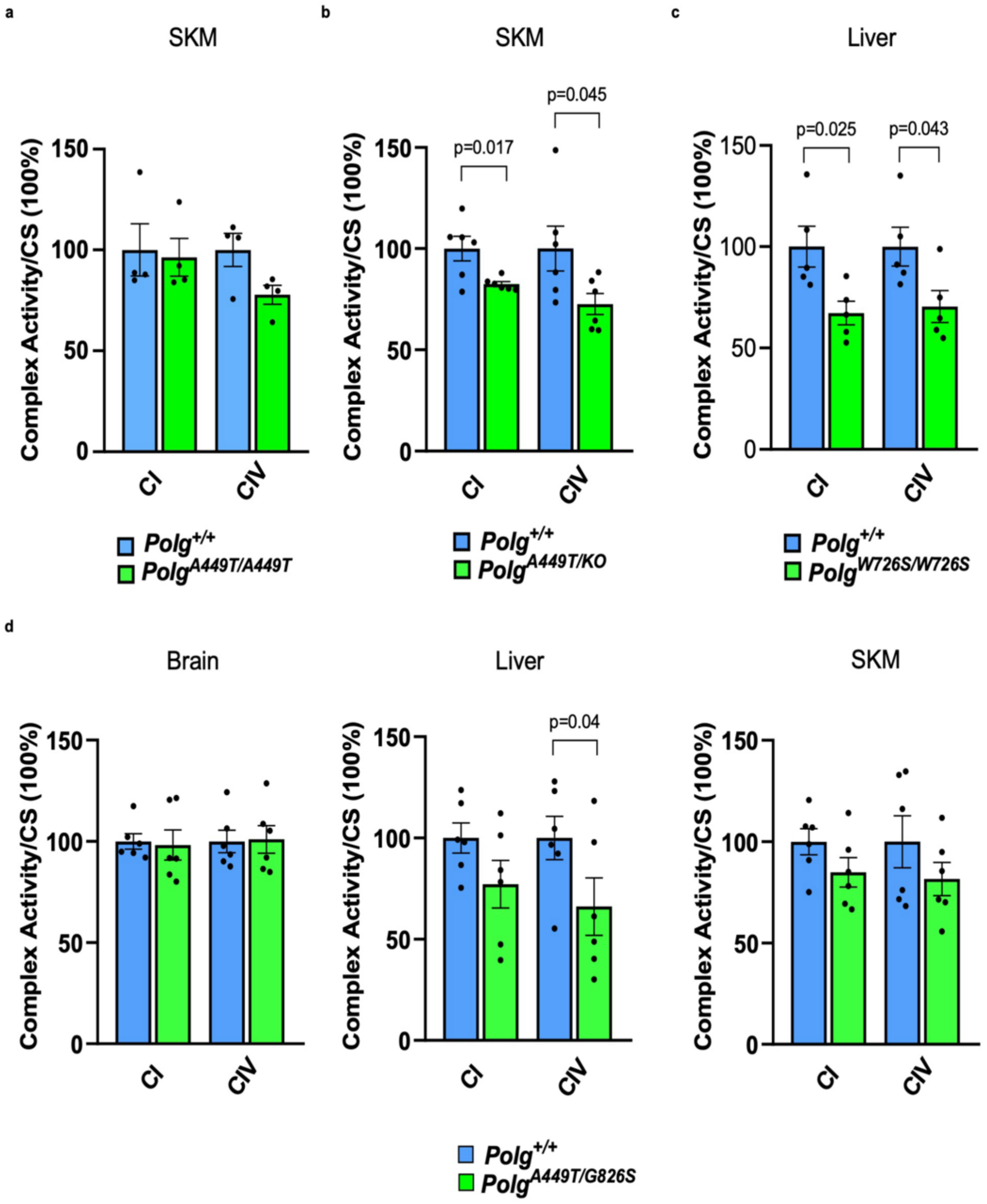

Supplementary Fig. 18. Analysis of OXPHOS activities.

Spectrophotometric activity of complex I (CI) and complex IV (CIV), normalized to citrate synthase (CS), in:

a. SKM of *Polg*$^{+/+}$ and *Polg*$^{A449T/A449T}$ mice (n = 4/genotype),

b. SKM of *Polg*$^{+/+}$ and *Polg*$^{A449T/KO}$ mice (n = 6/genotype),

c. Liver of *Polg*$^{+/+}$ and *Polg*$^{W726S/W726S}$ animals (n = 5/genotype),

d. Brain, liver, SKM of *Polg*$^{+/+}$ and *Polg*$^{A449T/G826S}$ mice (n = 6/genotype).

Both males and females were used. Data are presented as mean ±SEM. P-values were calculated by two tailed unpaired Student's t-test. Source data are provided as a Source Data file.

a

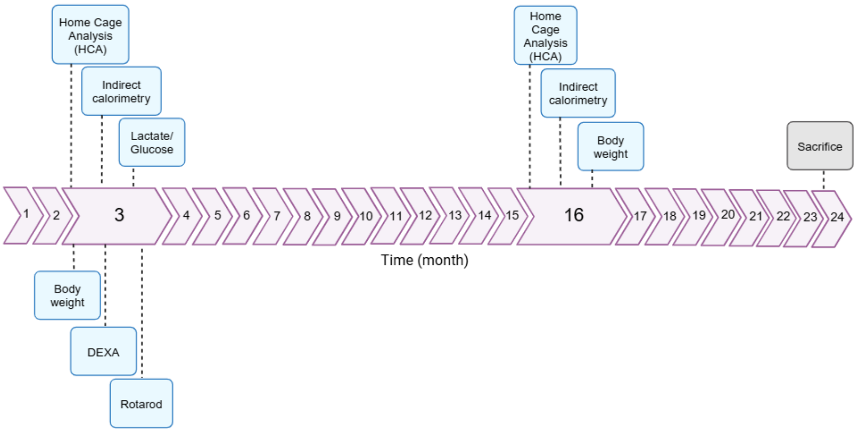

Home Cage Analysis (HCA)
Indirect calorimetry
Lactate/ Glucose
Body weight
DEXA
Rotarod
Home Cage Analysis (HCA)
Indirect calorimetry
Body weight
Sacrifice
1 2 3 4 5 6 7 8 9 10 11 12 13 14 15 16 17 18 19 20 21 22 23 24
Time (month)

b

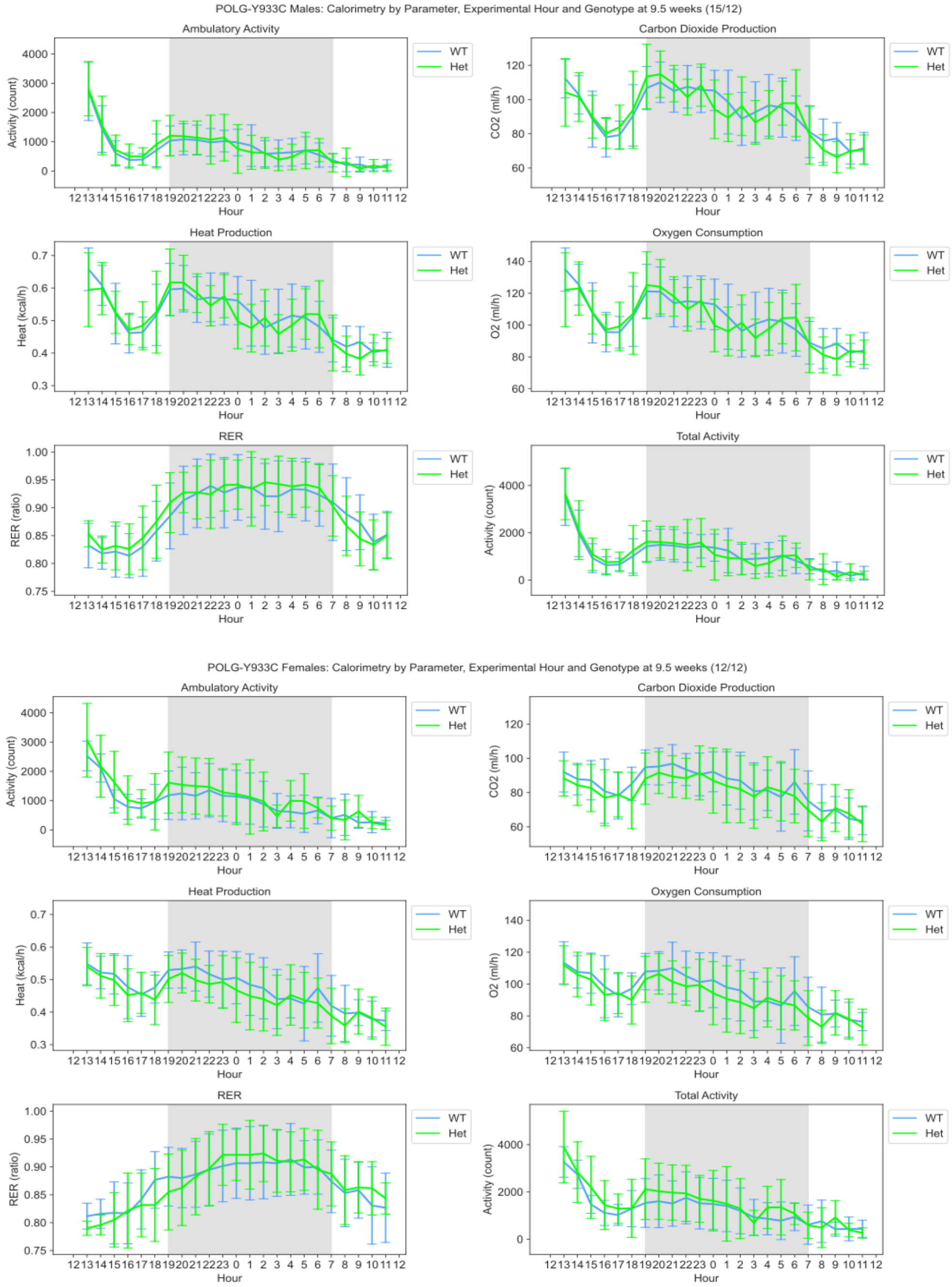

POLG-Y933C Males: Calorimetry by Parameter, Experimental Hour and Genotype at 9.5 weeks (15/12)
Ambulatory Activity
Carbon Dioxide Production
Heat Production
Oxygen Consumption
RER
Total Activity
Activity (count)
CO2 (ml/h)
Heat (kcal/h)
O2 (ml/h)
RER (ratio)
Hour
WT
Het
POLG-Y933C Females: Calorimetry by Parameter, Experimental Hour and Genotype at 9.5 weeks (12/12)
Ambulatory Activity
Carbon Dioxide Production
Heat Production
Oxygen Consumption
RER
Total Activity
Activity (count)
CO2 (ml/h)
Heat (kcal/h)
O2 (ml/h)
RER (ratio)
Hour
WT
Het

Supplementary Fig. 19. *In vivo* phenotypization of 3-month-old *Polg*$^{+/+}$ and *Polg*$^{+/Y933C}$ females and males.

a. Schematic overview of the *in vivo* pipeline.

b. Indirect calorimetry for males (*n* = 15 WT and 12 *Polg*$^{+/Y933C}$ (Het); upper panel) and females (*n* = 12 WT and 12 *Polg*$^{+/Y933C}$ (Het); lower panels). The shaded grey background represents the dark period, the white background the light period. Ambulatory activity, CO2 production, O2 consumption, Heat production, RER, and total movements measured with CLAMS are shown. See Methods for details. Source data are provided as a Source Data file.

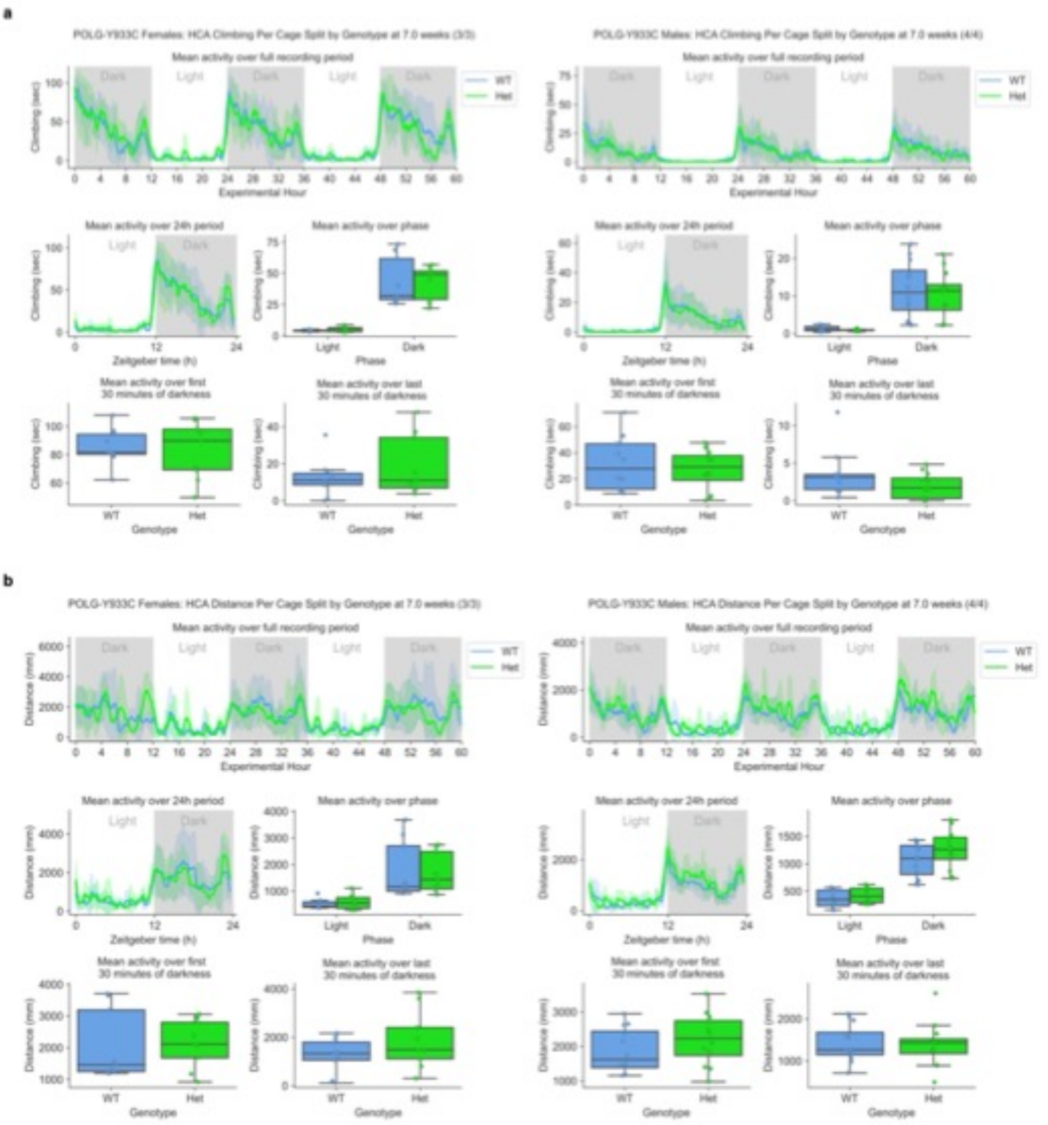

Supplementary Fig. 20. HCA analysis of 3-month-old *Polg*$^{+/+}$ and *Polg*$^{+/Y933C}$ females and males.

a. Climbing activity for females (*n* = 3 WT and 3 *Polg*$^{+/Y933C}$ (Het); left panel) and males (*n* = 4 WT and 4 *Polg*$^{+/Y933C}$ (Het); right panel).

b. Distance for females (*n* = 3 WT and 3 *Polg*$^{+/Y933C}$ (Het); left panel) and males (*n* = 4 WT and 4 *Polg*$^{+/Y933C}$ (Het); right panel). Source data are provided as a Source Data file.

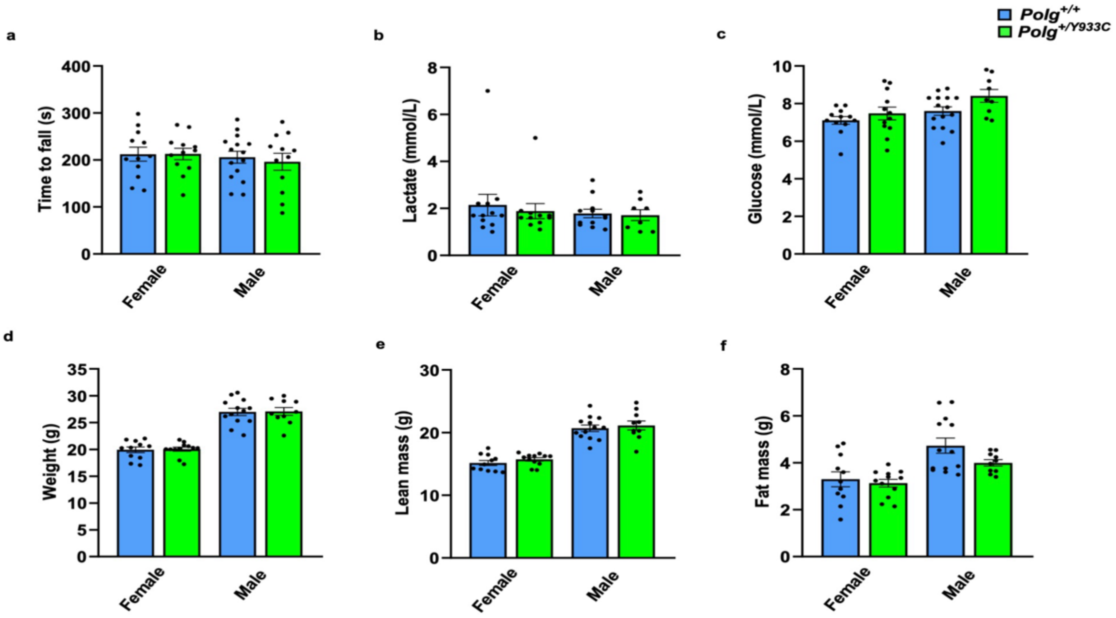

Supplementary Fig. 21. Metabolic analysis of 3-month-old *Polg*$^{+/+}$ and *Polg*$^{+/Y933C}$ females and males.

a. Rotarod performance (females *n* = 12/genotype; males *n* = 15 WT and 12 *Polg*$^{+/Y933C}$).

b. Lactate levels (females *n* = 12 WT and 12 *Polg*$^{+/Y933C}$; males *n* = 12 WT and 8 *Polg*$^{+/Y933C}$).

c. Glucose levels (females *n* = 12/genotype; males *n* = 15 WT and 9 *Polg*$^{+/Y933C}$).

d. Body weights (females *n* = 11 WT and 12 *Polg*$^{+/Y933C}$; males *n* = 13 WT and 10 *Polg*$^{+/Y933C}$).

e-f. Lean and fat mass (females *n* = 11 WT and 12 *Polg*$^{+/Y933C}$; males *n* = 13 WT and 10 *Polg*$^{+/Y933C}$).

Source data are provided as a Source Data file.

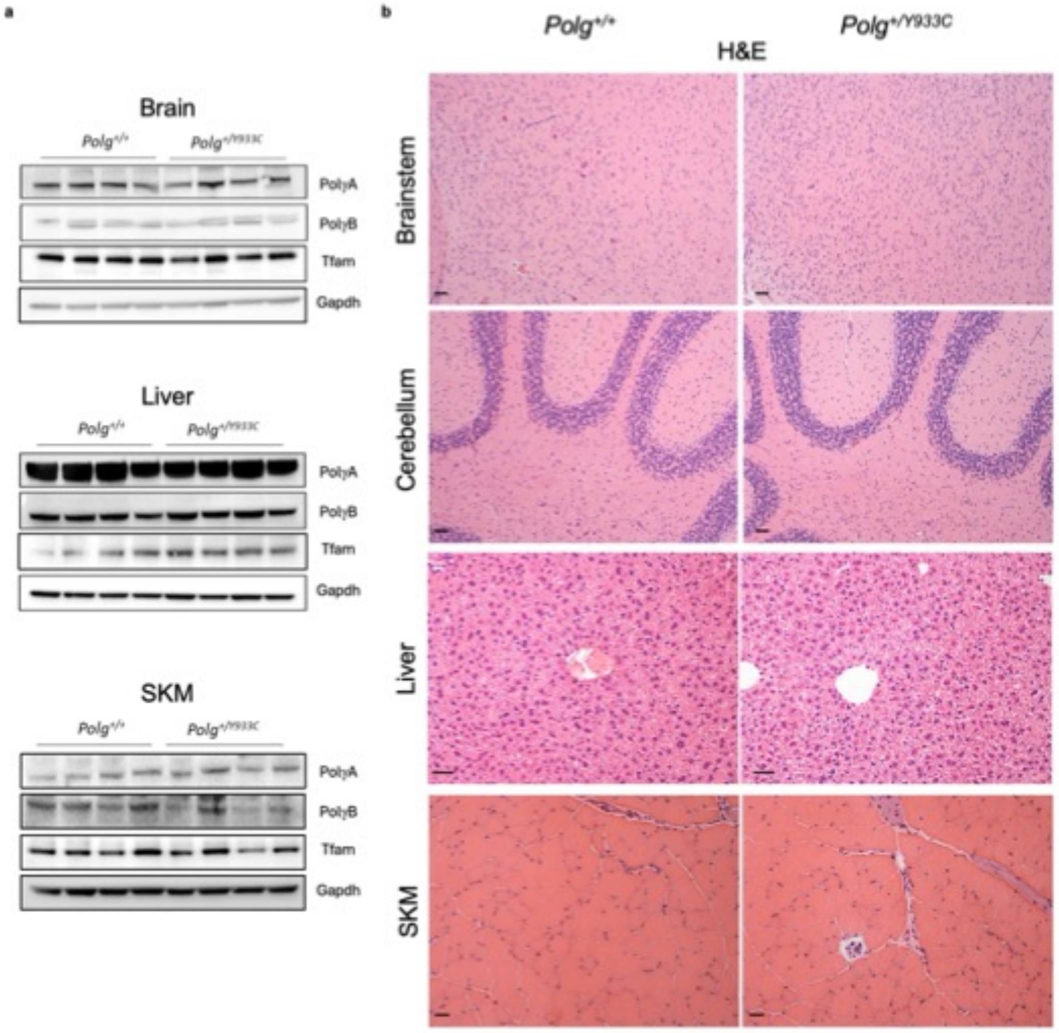

Supplementary Fig. 22. Postmortem analysis of 3-month-old *Polg*$^{+/+}$ and *Polg*$^{+/Y933C}$ mice.

a. Western blot analysis of the mitochondrial replisome in brain, liver and skeletal muscle (n = 4/genotype). Both males and females were used.

b. H&E staining was performed in brainstem, cerebellum, liver and SKM samples of *Polg*$^{+/+}$ (left) and *Polg*$^{+/Y933C}$ (right) animals. H&E staining did not reveal any obvious morphological alterations. Scale bars: brainstem and cerebellum 50 µm, liver 40 µm; SKM 20 µm. (n = 3/genotype). Both males and females were used.

Source data are provided as a Source Data file.

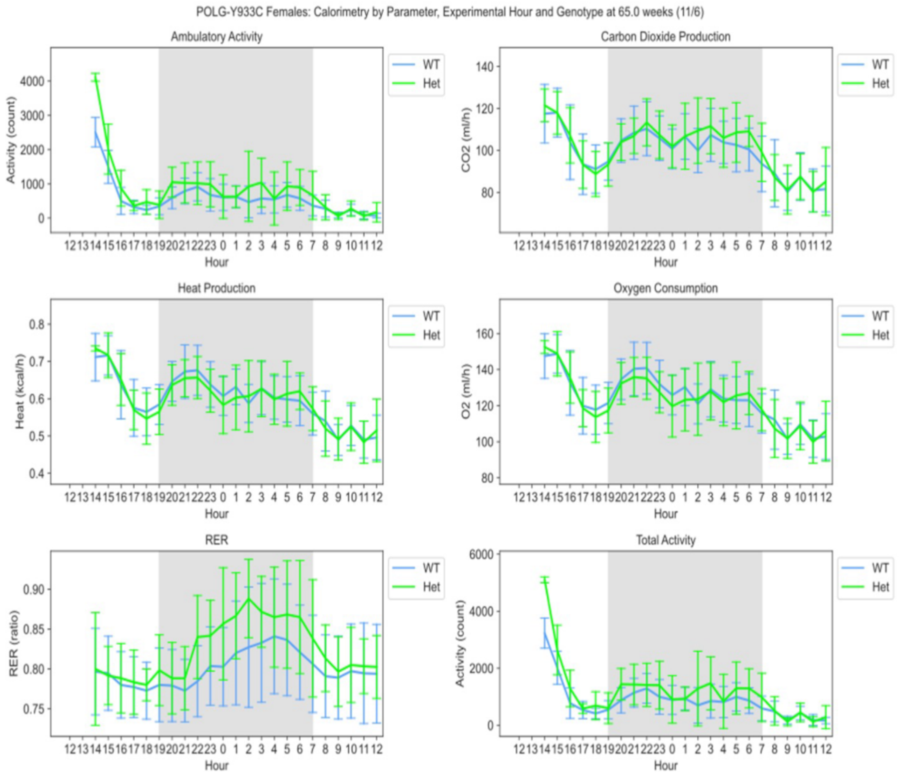
POLG-Y933C Females: Calorimetry by Parameter, Experimental Hour and Genotype at 65.0 weeks (11/6)
Ambulatory Activity
Carbon Dioxide Production
Heat Production
Oxygen Consumption
RER
Total Activity
Activity (count)
CO2 (ml/h)
Heat (kcal/h)
O2 (ml/h)
RER (ratio)
Hour
WT
Het

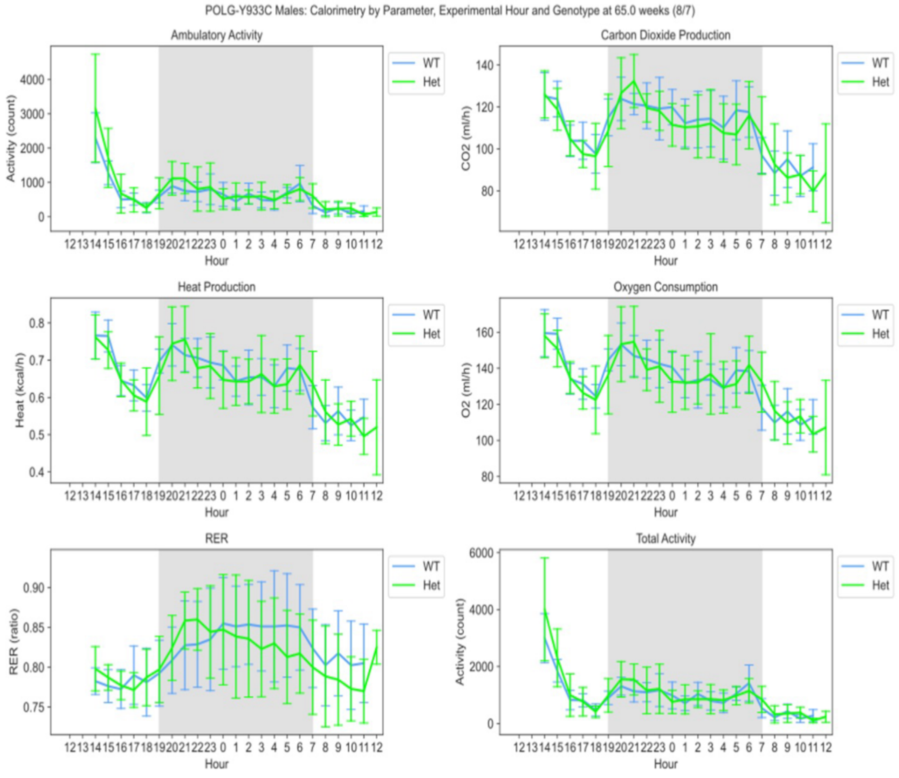
POLG-Y933C Males: Calorimetry by Parameter, Experimental Hour and Genotype at 65.0 weeks (8/7)
Ambulatory Activity
Carbon Dioxide Production
Heat Production
Oxygen Consumption
RER
Total Activity
Activity (count)
CO2 (ml/h)
Heat (kcal/h)
O2 (ml/h)
RER (ratio)
Hour
WT
Het

Supplementary Fig. 23. Indirect calorimetry of 16-month-old *Polg*$^{+/+}$ and *Polg*$^{+/Y933C}$ females and males.

Indirect calorimetry for females (*n* = 11 WT and 6 *Polg*$^{+/Y933C}$ (Het); upper panel) and males (*n* = 6 WT and 7 *Polg*$^{+/Y933C}$ (Het); lower panel). The shaded grey background represents the dark period, the white background the light period. Ambulatory activity, CO2 production, O2 consumption, Heat production, RER, and total movements measured with CLAMS are shown. See Methods for details. Source data are provided as a Source Data file.

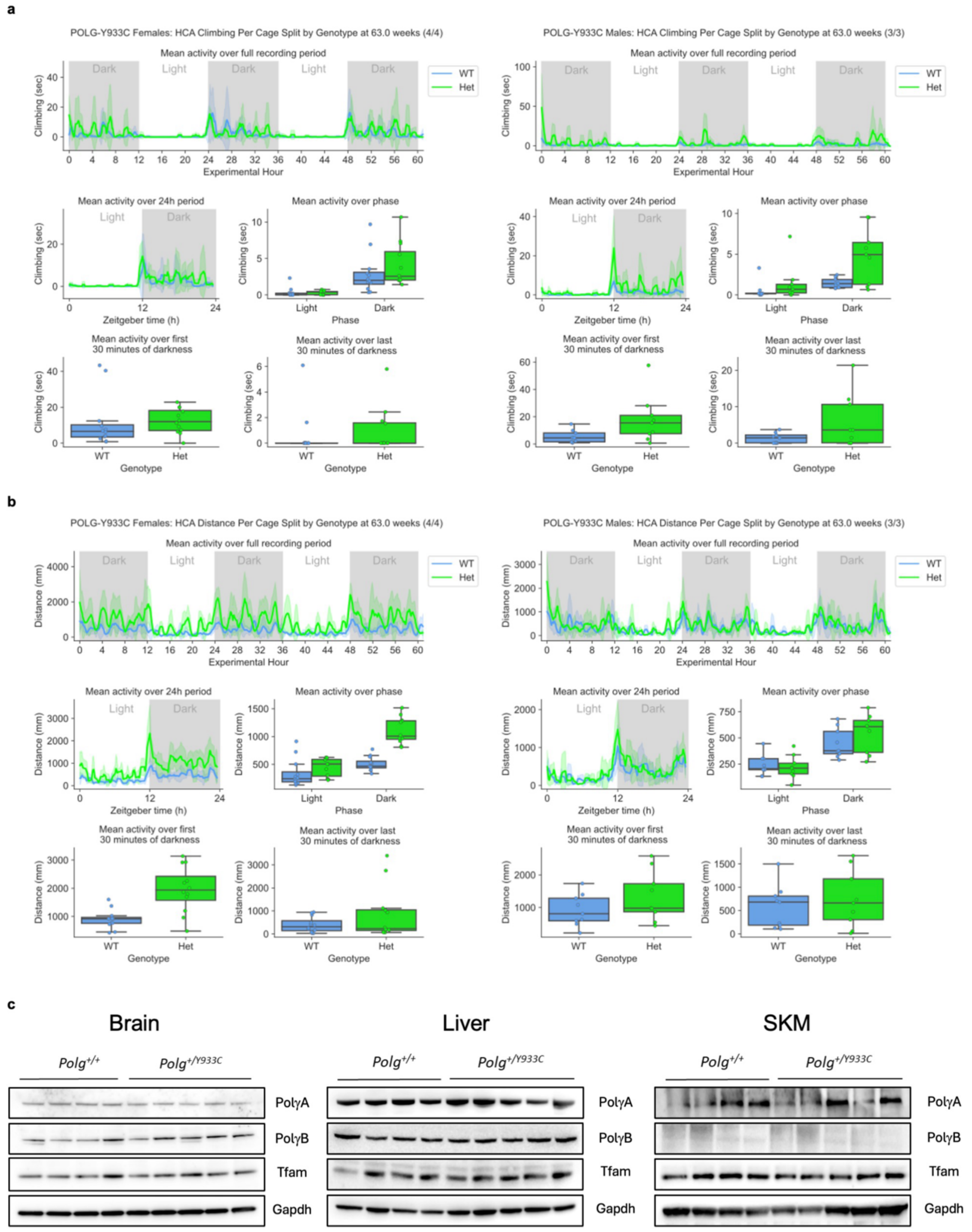

Supplementary Fig. 24. Analysis of old *Polg*$^{+/+}$ and *Polg*$^{+/Y933C}$ females and males

a. HCA climbing activity at 16 months of age for females (*n* = 4 WT and 4 *Polg*$^{+/Y933C}$ (Het); left panel) and males (*n* = 3 WT and 3 *Polg*$^{+/Y933C}$ (Het); right panel).

b. HCA distance at 16 months of age for females (*n* = 4 WT and 4 *Polg*$^{+/Y933C}$ (Het); left panel) and males (*n* = 3 WT and 3 *Polg*$^{+/Y933C}$ (Het); right panel).

c. Western blot analysis of the mitochondrial replisome in brain, liver and skeletal muscle from 24-month-old mice (n = 4 WT and 5 $Polg^{+/Y933C}$). Both males and females were used. Source data are provided as a Source Data file.

Supplementary table 1. Cryo-EM data collection, refinement and validation statistics.

	Mouse POLγ			Mouse POLγA<br>Human POLγB			Human POLγA<br>Mouse POLγB	
	R-conformer<br>(EMDB-51109)<br>(PDB 9G74)	I-conformer<br>(EMDB-51110)<br>(PDB 9G75)	E-conformer<br>(EMDB-51114)<br>(PDB 9G77)	R-conformer<br>(EMDB-52815)<br>(PDB 9IBX)	E-conformer<br>Human-like<br>(EMDB-52819)<br>(PDB 9IBZ)	E-conformer<br>Mouse-like<br>(EMDB-52823)<br>(PDB 9IC0)	R-conformer<br>(EMDB-52824)<br>(PDB 9IC1)	E-conformer<br>Mouse-like<br>(EMDB-52828)<br>(PDB 9IC3)
**Data collection and processing**								
Magnification	105,000	105,000	105,000	105,000	105,000	105,000	105,000	105,000
Voltage (kV)	300	300	300	300	300	300	300	300
Electron exposure (e^-/Å^2)	40	40	40	40	40	40	40	40
Defocus range (μm)	0.8-2.2	0.8-2.2	0.8-2.2	0.8-2.2	0.8-2.2	0.8-2.2	0.8-2.2	0.8-2.2
Pixel size (Å)	0.825	0.825	0.825	0.828	0.828	0.828	0.828	0.828
Symmetry imposed	C1	C1	C1	C1	C1	C1	C1	C1
Initial particle images (no.)	10,917,170	10,917,170	10,917,170	6,368,562	6,368,562	6,368,562	9,142,462	9,142,462
Final particle images (no.)	832,556	275,211	930,574	713,960	370,910	212,286	448,270	516,159
Map resolution (Å)	2.75	2.98	2.87	2.54	3.08	3.24	2.73	2.96
FSC threshold	0.143	0.143	0.143	0.143	0.143	0.143	0.143	0.143
Map resolution range (Å)	2.3-31.5	2.6-41.0	2.5-38.7	2.2-4.9	2.7-44.5	2.8-39.2	2.4-29.5	2.5-43.4
**Refinement**								
Initial model used (PDB code)	4ZTZ	4ZTZ	4ZTZ	4ZTZ	4ZTZ	4ZTZ	4ZTZ	4ZTZ/8D42
Model resolution (Å)	3.1	3.4	3.0	3.2	3.5	3.7	3.4	3.4
FSC threshold	0.5	0.5	0.5	0.5	0.5	0.5	0.5	0.5
Model composition								
Non-hydrogen atoms	14,683	13,780	14,790	14,129	14,339	13,987	14,182	13,841
Protein/DNA residues	1,741/38	1,634/38	1,742/41	1661/38	1,680/41	1636/41	1682/38	1,627/41
Ligands	2	0	3	2	3	3	2	4
B factors (Å^2)								
Protein	58.79	50.11	67.36	118.96	130.30	73.63	69.90	42.28
DNA	76.04	137.47	114.76	150.28	222.85	149.57	107.36	115.44
Ligands	59.51	-	102.71	138.07	172.96	95.31	74.30	66.42
R.m.s. deviations								
Bond lengths (Å)	0.003	0.003	0.003	0.004	0.002	0.003	0.003	0.005
Bond angles (°)	0.485	0.515	0.519	0.549	0.444	0.519	0.509	0.547
Validation								
MolProbity score	1.48	1.55	1.48	1.38	1.42	1.60	1.31	1.69
Clashscore	7.50	8.40	7.18	6.32	6.37	7.89	5.64	9.14
Poor rotamers (%)	0.53	0.28	0.40	0.55	0.68	0.56	0.62	0.85
Ramachandran plot								
Favored (%)	97.67	97.56	97.61	97.85	97.70	97.01	98.12	96.78
Allowed (%)	2.33	2.44	2.39	0.15	2.30	2.99	1.88	3.22
Disallowed (%)	0.00	0.00	0.00	0.00	0.00	0.00	0.00	0.00

Supplementary table 2. Analysis of the distribution of *PolgY933C* genotypes.

**Genotype**	**N. of animals**	**%**
Polg$^{Y933C/Y933C}$	4	10,53
Polg$^{Y933C/+}$	28	73,68
Polg$^{+/+}$	6	15,79
**Total**	38	

Supplementary Table 3. gRNAs, donor oligonucleotides, and primers used in this study.

gRNA_Y933C	ATGGCCGCATCTATGGGGCT
ssDNA_HDR_Y933C	GGGCAGGAAGAGCAGAGGCACTGATCTGCACAGCAAGACAGCTGCCACTGTGGGCATCAGCCGAG AGCATGCCAAAATCTTCAACTATGGCCGCATCTGTGGGGCTGGCCAGTCCTTTGCTGAGCGCCTACT GATGCAGTTCAACCACAGGCTCACAAGGCAGGAGGCA
gRNA_G826S	GGTGCCAGCAGTCACCACCT
ssDNA_HDR_G826S	TGAAAGAGGAAGAAGAGAATCCAGGAACAGAGAGGACATATACCCGAGCATTACTGGCAGTGAGCCACGT GGGCTCCACAGCTCGACGGGTGATGGTGCTAGCAGTCACCACCTGTGGTAGGATGGCCCCATAGTGGCC TTCCTCATCGAAGCTGGGGTGCCTGGAATAGGGAGAAGCAAAGAAGTGTCAGCAGGCTTGT
LongR_mtDNA_Fw	GAGGTGATGTTTTTGGTAAACAGGCGGGGT
LongR_mtDNA_Rv	GGTTCGTTTGTTCAACGATTAAAGTCCTACGTG
A449T_Fw	GTTGTCCCTGTCTTCCTCCA
A449T_Rv	AAGCTTCCCACCTTCCTGAT
Y933C_Fw	GCCTTTGGCTGGATGACTCT
Y933C_Rv	CAGCGCCTGTCTATCTGCTT
G826S_Fw	CAGATGGTGGTATGGCTCCC
G826S_Rv	AGGACCACCTAGAAGCCCAA
KO_Fw1	CTTCGTCGATCGACCTCGAATAAC
KO_Fw2	GGATGGGCAGGAACAGTTAG
KO_Rv	CTGCCATTCACCTTACCC
W726S_Fw	AGGGAACCTGTGACCATGCTCTCC
W726S_Rv	GGTAACCCCACCAGGATGCCTC
qPCR_mCoI_Fw	TGCTAGCCGCAGGCATTACT
qPCR_mCoI_Rv	CGGGATCAAAGAAAGTTGTGTTT
qPCR_RnaseP_Fw	GCCTACACTGGAGTCGTGCTACT
qPCR_RnaseP_Rv	CTGACCACACGAGCTGGTAGAA
