# Supplementary material for: Modelling POLG mutations in mice unravels a critical role of POLγΒ in regulating phenotypic severity
Source: Nat Commun. 2025 May 23;16:4782. doi: 10.1038/s41467-025-60059-y (PMC12098916; doi:10.1038/s41467-025-60059-y)
Supplement: Supplementary file 2 — Reporting Summary [file 41467_2025_60059_MOESM2_ESM.pdf]

nature portfolio

Corresponding author(s): Xuefeng Zhu, Maria Falkenberg, Carlo Viscomi

Last updated by author(s): 25/3/25

Reporting Summary

Nature Portfolio wishes to improve the reproducibility of the work that we publish. This form provides structure for consistency and transparency in reporting. For further information on Nature Portfolio policies, see our Editorial Policies and the Editorial Policy Checklist.

Statistics

For all statistical analyses, confirm that the following items are present in the figure legend, table legend, main text, or Methods section.

n/a	Confirmed	
☐	☒	The exact sample size (*n*) for each experimental group/condition, given as a discrete number and unit of measurement
☐	☒	A statement on whether measurements were taken from distinct samples or whether the same sample was measured repeatedly
☐	☒	The statistical test(s) used AND whether they are one- or two-sided<br>*Only common tests should be described solely by name; describe more complex techniques in the Methods section.*
☒	☐	A description of all covariates tested
☒	☐	A description of any assumptions or corrections, such as tests of normality and adjustment for multiple comparisons
☐	☒	A full description of the statistical parameters including central tendency (e.g. means) or other basic estimates (e.g. regression coefficient) AND variation (e.g. standard deviation) or associated estimates of uncertainty (e.g. confidence intervals)
☒	☐	For null hypothesis testing, the test statistic (e.g. *F*, *t*, *r*) with confidence intervals, effect sizes, degrees of freedom and *P* value noted<br>*Give P values as exact values whenever suitable.*
☒	☐	For Bayesian analysis, information on the choice of priors and Markov chain Monte Carlo settings
☒	☐	For hierarchical and complex designs, identification of the appropriate level for tests and full reporting of outcomes
☒	☐	Estimates of effect sizes (e.g. Cohen's *d*, Pearson's *r*), indicating how they were calculated

Our web collection on statistics for biologists contains articles on many of the points above.

Software and code

Policy information about availability of computer code

Data collection	BMG PHERAstar microtiter plate reader control software(v5.70 R6); BioRad CFX Maestro real time software(v2.3);
Data analysis	In addition to above mentioned software,the following softwares were used in this study: BMG MARS data analysis software(V4.01 R2); Fujifilm Multi Gauge software(V3.1); BioRad Image lab software(v6.1);cryoSPARC (v4.3.1);DeepEMhancer (v0.14); Coot (v.0.9.8.1) and ISOLDE (v.1.4);PHENIX (v.1.20.);UCSF ChimeraX(v1.4); Graphpad Prism Software (v9.0 and 10.0)

For manuscripts utilizing custom algorithms or software that are central to the research but not yet described in published literature, software must be made available to editors and reviewers. We strongly encourage code deposition in a community repository (e.g. GitHub). See the Nature Portfolio guidelines for submitting code & software for further information.

Data

Policy information about availability of data

All manuscripts must include a data availability statement. This statement should provide the following information, where applicable:

- Accession codes, unique identifiers, or web links for publicly available datasets
- A description of any restrictions on data availability
- For clinical datasets or third party data, please ensure that the statement adheres to our policy

The atomic models and cryo-EM density maps have been deposited in the Protein Data Bank and the Electron Microscopy Data Bank under the following accession codes: Mouse R conformer (9G74, EMD-51109), Mouse I conformer (9G75, EMD-51110) and the Mouse E conformer (9G77, EMD-51114). Accession codes for consensus and local refinement maps of the mouse E conformer are EMD-51111 (consensus), EMD-51112 (local refinement of mPolA and DNA) and EMD-51113

(local refinement of mPolB subunits). The chimeric complex (mAhB) was assigned the following accession codes: R conformer (9IBX, EMD-52815), “human-like” E conformer (9IBZ, EMD-52819) and “mouse-like” E conformer (9IC0, EMD-52823). Accession codes for consensus and local refinement maps of the mAhB “human-like” E conformer are EMD-52816 (consensus), EMD-52817 (local refinement of mPolA and DNA) and EMD-52818 (local refinement of mPolB subunits). Accession codes for consensus and local refinement maps of the mAhB “mouse-like” E conformer are EMD-52820 (consensus), EMD-52821 (local refinement of mPolA and DNA) and EMD-52822 (local refinement of mPolB subunits). The chimeric complex (hAmB) was given the following accession codes: R conformer (9IC1, EMD-52824) and “mouse-like” E conformer (9IC3, EMD-52828). Accession codes for consensus and local refinement maps of the hAmB “mouse-like” E conformer are EMD-52825 (consensus), EMD-52826 (local refinement of mPolA and DNA) and EMD-52827 (local refinement of mPolB subunits).

Research involving human participants, their data, or biological material

Policy information about studies with human participants or human data. See also policy information about sex, gender (identity/presentation), and sexual orientation and race, ethnicity and racism.

Reporting on sex and gender	n/a
Reporting on race, ethnicity, or other socially relevant groupings	n/a
Population characteristics	n/a
Recruitment	n/a
Ethics oversight	n/a

Note that full information on the approval of the study protocol must also be provided in the manuscript.

Field-specific reporting

Please select the one below that is the best fit for your research. If you are not sure, read the appropriate sections before making your selection.

[x] Life sciences [ ] Behavioural & social sciences [ ] Ecological, evolutionary & environmental sciences

For a reference copy of the document with all sections, see nature.com/documents/nr-reporting-summary-flat.pdf

Life sciences study design

All studies must disclose on these points even when the disclosure is negative.

Sample size	No statistical method was used to predetermine sample size, and group sizes were determined based on the results of preliminary experiments.
Data exclusions	No data were excluded from the analysis.
Replication	The animals came from different litters and the results were reproducible.
Randomization	No randomization was used as the mice were allocated to the experimental groups on the basis of their genotypes.
Blinding	No blinding to the operator was used.

Reporting for specific materials, systems and methods

We require information from authors about some types of materials, experimental systems and methods used in many studies. Here, indicate whether each material, system or method listed is relevant to your study. If you are not sure if a list item applies to your research, read the appropriate section before selecting a response.

Materials & experimental systems

n/a	Involved in the study	
[ ]	[x]	Antibodies
[x]	[ ]	Eukaryotic cell lines
[x]	[ ]	Palaeontology and archaeology
[ ]	[x]	Animals and other organisms
[x]	[ ]	Clinical data
[x]	[ ]	Dual use research of concern
[x]	[ ]	Plants

Methods

n/a	Involved in the study	
[x]	[ ]	ChIP-seq
[x]	[ ]	Flow cytometry
[x]	[ ]	MRI-based neuroimaging

Antibodies

Antibodies used	anti-POLγA (1:500) was from Cell Signaling (D1Y6R), anti-POLγB (1:500) was from Novus (NBP2-94064), anti-GAPDH (1:5000) was from GeneTex (GTX627408), anti-TFAM (1:2000) was from Abcam (ab131607). Secondary antibodies were from Promega (catalog nos. W4011 [rabbit], and W4021 [mouse]).
Validation	anti-POLγA: Cell Signaling (D1Y6R), has been referenced in 9 publications. https://www.cellsignal.com/products/primary-antibodies/dna-polymerase-g-d1y6r-rabbit-mab/13609?srsltid=AfmBOoqT3Wta072VygKIPpzgLFJm5IXiouwG3O8Aaa-bmZKSLB2Sn2Qu<br>anti-POLγB: Novus (NBP2-94064), has not been reported in publication. This antibody recognizes a single band at 55 KDain all mouse tissues, although two bands were detected in the brain as pèreviously reported using a different antibody (doi: 10.1093/nar/gkab282).<br>anti-GAPDH: GeneTex (GTX627408) has been cited in 478 publications. https://www.genetex.com/Product/Detail/GAPDH-antibody-GT239/GTX627408?srsltid=AfmBOooCMZ8xSNxpG--kQEMgL3FZDya8W_Uwz89V4qFmkfTMi2yBWszt<br>anti-TFAM: Abcam (ab131607) has been cited in 158 publications. https://www.abcam.com/en-us/products/primary-antibodies/mttfa-antibody-mitochondrial-marker-ab131607

Animals and other research organisms

Policy information about studies involving animals; ARRIVE guidelines recommended for reporting animal research, and Sex and Gender in Research

Laboratory animals	Males and females were used from the following strains: PolgA449T/A449T (FVB/J) , PolgW726S/W726S (C57BL6/J), Polg+/G826S (C57BL6/J), Polg+/KO (FVB/J) ,Polg+/Y933C (C57BL6/J), PolgA449T/KO (FVBJ), PolgA449T/G826S (FVB/J x C57BL6/J), PolgW726S/G826S (C57BL6/J), PolgA449T/W726S (FVB/J x C57BL6/J). For outbred mice wild type littermates were used as controls. The animals were maintained in a temperature- and humidity-controlled animal care facility with a 12-h light/12-h dark cycle and free access to water and food, and they were monitored weekly to examine body condition, weight and general health. The mice were sacrificed by cervical dislocation for subsequent analysis.
Wild animals	n/a
Reporting on sex	We pooled males and females for the molecular analysis wehere no differences were observed. We reported separate analysis for the behavioural studies where we found significant differences.
Field-collected samples	n/a
Ethics oversight	All animal experiments were carried out in accordance with EU Directive 2010/63/EU (authorization from the Italian Miinistry of Health: PR474/2021)

Note that full information on the approval of the study protocol must also be provided in the manuscript.

Plants

Seed stocks	n/a
Novel plant genotypes	n/a
Authentication	n/a
